# A Robust α-l-Fucosidase from *Prevotella nigrescens* for Glycoengineering Therapeutic Antibodies

**DOI:** 10.1021/acschembio.4c00196

**Published:** 2024-06-24

**Authors:** Mu-Rong Kao, Tzu-Hsuan Ma, Hsiang-Yu Chou, Shu-Chieh Chang, Lin-Chen Cheng, Kuo-Shiang Liao, Jiun-Jie Shie, Philip J. Harris, Chi-Huey Wong, Yves S. Y. Hsieh

**Affiliations:** † School of Pharmacy, College of Pharmacy, Taipei Medical University, No. 250 Wuxing Street, Taipei 11031, Taiwan; ‡ Genomics Research Center, Academia Sinica, No. 128 Academia Road, Section 2, Nankang District, Taipei 115201, Taiwan; § Division of Glycoscience, Department of Chemistry, School of Engineering Sciences in Chemistry, Biotechnology and Health, Royal Institute of Technology (KTH), AlbaNova University Centre, Stockholm SE-10691, Sweden; ∥ Institute of Chemistry, Academia Sinica, No. 128 Academia Road, Section 2, Nankang District, Taipei 115201, Taiwan; ⊥ School of Biological Sciences, The University of Auckland, Auckland Mail Centre, Private Bag 92019, Auckland 1142, New Zealand; # Department of Chemistry, The Scripps Research Institute, 10550 North Torrey Pines Road, La Jolla, California 92037, United States

## Abstract

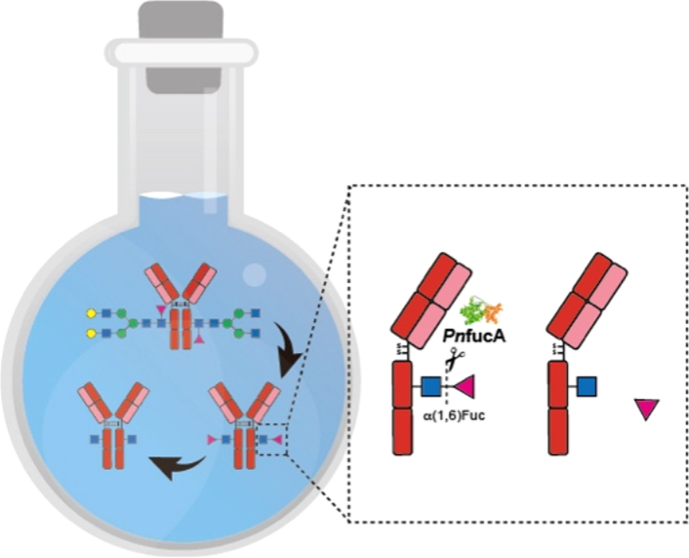

Eliminating the core fucose from the *N*-glycans of the Fc antibody segment by pathway engineering or enzymatic methods has been shown to enhance the potency of therapeutic antibodies, especially in the context of antibody-dependent cytotoxicity (ADCC). However, there is a significant challenge due to the limited defucosylation efficiency of commercially available α-l-fucosidases. In this study, we report a unique α-l-fucosidase (*Pn*fucA) from the bacterium *Prevotella nigrescens* that has a low sequence identity compared with all other known α-l-fucosidases and is highly reactive toward a core disaccharide substrate with fucose α(1,3)-, α (1,4)-and α(1,6)-linked to GlcNAc, and is less reactive toward the Fuc-α(1,2)-Gal on the terminal trisaccharide of the oligosaccharide Globo H (Bb3). The kinetic properties of the enzyme, such as its *K*_m_ and *k*_cat_, were determined and the optimized expression of *Pn*fucA gave a yield exceeding 30 mg/L. The recombinant enzyme retained its full activity even after being incubated for 6 h at 37 °C. Moreover, it retained 92 and 87% of its activity after freezing and freeze-drying treatments, respectively, for over 28 days. In a representative glycoengineering of adalimumab (Humira), *Pn*fucA showed remarkable hydrolytic efficiency in cleaving the α(1,6)-linked core fucose from FucGlcNAc on the antibody with a quantitative yield. This enabled the seamless incorporation of biantennary sialylglycans by Endo-S2 D184 M in a one-pot fashion to yield adalimumab in a homogeneous afucosylated glycoform with an improved binding affinity toward Fcγ receptor IIIa.

## Introduction

Fucosylation is a post-translational modification that plays a vital role in modulating the functions of glycolipids and glycoproteins.^1^ It is intricately associated with ABO blood typing,^2^ cancer progression,^3^ onset of inflammatory responses,^4^ and the efficacy of antibody effector functions.^5^ The *N*-glycan of the antibody fragment crystallizable (Fc) domain that contains the core l-fucose (l-Fuc) linked to the innermost *N*-acetylglucosamine (GlcNAc) residue can modulate the interaction of the antibody with the Fc receptor FcγRIIIa on natural killer (NK) cells and peripheral blood monocytes (PBMC). The core fucose interferes with these interactions, resulting in a decreased antibody-dependent cellular cytotoxicity (ADCC),^6^ an important immunological process that involves the destruction of target cells, such as infected or cancerous cells.

Strategies for enhancing the ADCC efficacy of therapeutic monoclonal antibodies (mAbs) by producing afucosylated mAbs have been developed, particularly in the context of trastuzumab (anti-Her2/neu)^7,8^ and rituximab (anti-CD20).^9,10^ One strategy entails the use of 2-fluoro-l-fucose (2F-Fuc) analogues as inhibitors that can substantially decrease the incorporation of core fucose.^11,12^ An alternative method consists of engineering CHO cell lines with the *FUT8* gene knockout to produce afucosylated mAbs.^13,14^ However, the absence of *FUT8* also markedly reduces the activity of sialyltransferases and other glycosyltransferases.^15^

The majority of therapeutic mAbs currently available on the market have a high degree of fucosylation (over 90%), a characteristic primarily attributed to the inherent properties of the host cell lines and the imperative need to preserve cellular functions.^5^ It has also been reported that low fucose IgGs showed at least a 50-fold increase in ADCC activity compared with the fully fucosylated counterpart.^16−18^ Therefore, to create a therapeutic antibody with maximum ADCC activity, it is crucial to establish a robust process that completely eliminates the core fucose.

**Scheme 1:**
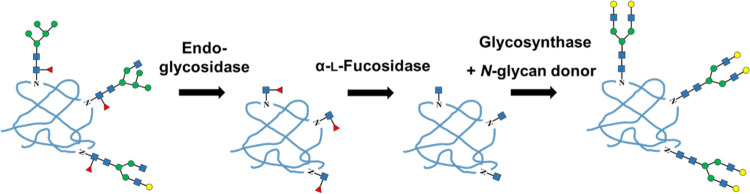
Glycoengineering of *N*-Linked Glycoprotein

Until recently, the following glycoengineering approach has been used as an effective strategy for generating afucosylated antibodies.^9,19^ It is an efficient *in vitro* method to produce homogeneous antibody glycoforms with the desired *N*-glycan structure at Asn 297 to modulate effector functions.^20−23^ The method begins with the removal of heterogeneous *N*-glycans using an endo-β-*N*-acetylglucosaminidase, commonly referred to as “Endo”^24−26^ (Scheme 1). Following this step, the critical enzyme α-l-fucosidase is used to remove core fucose residues. Using highly efficient α-l-fucosidases is crucial for optimizing mAb Fc glycoengineering within the shortest possible reaction times, thereby ensuring the preservation of full antibody activity. Finally, transglycosylation is carried out using a glycosynthase, for putting on desired glycan moieties.^9,27^

Microbial α-l-fucosidases occur in two glycoside hydrolase families: 29 (GH29) and 95 (GH 95),^28^ with the GH29 enzymes occurring in both subfamily A (GH29A) and subfamily B (GH29B). The subfamily GH29A enzymes are recognized for having broad substrate specificities, acting on α(1,2)-, (1,3)-, (1,4)-, and (1,6)-linked fucosylated substrates. In contrast, the GH29B enzymes show glycosidic linkage selectivity, primarily targeting α(1,3)- and (1,4)-linked substrates. Our objective of this study was to identify a GH29A enzyme with exceptional efficiency in hydrolyzing the α(1,6)-linked core fucose in the Fc glycan, a pivotal step in the production of afucosylated monoclonal antibodies.

So far, only a few α-l-fucosidases have been described that show a pronounced substrate specificity for the core fucose in *N*-linked glycoproteins.^9,27,29,30^ For example, the *Lactobacillus casei* α-l-fucosidase AlfC has a clear preference for the FucGlcNAc disaccharide (FN), displaying high hydrolytic activity for the α(1,6)-linked FucGlcNAc disaccharide (6FN). This enzyme showed only moderate activity on the α(1,3)- and α(1,4)-linked FucGlcNAc disaccharides (3FN and 4FN)^31^ and has been used as the defucosylation enzyme for glycoengineering antibodies.^9,27^ Another enzyme, this time from *Bacteroides fragitis* (BfFucH), has also been shown to have preferred substrate activity for the efficient defucosylation of core fucose in homogeneous antibody production.^29^ In the present study, we report a unique α-l-fucosidase (*Pn*fucA) from the bacterium *Prevotella nigrescens* that has a low level of sequence identity compared with most α-l-fucosidases characterized so far (Table 1). Its efficiency in targeting the core fucose variants 3FN, 4FN, and 6FN is highlighted. In addition, this enzyme exhibits a significant synergy with glycosynthase in one-pot glycoengineering of adalimumab in a quantitative yield. The discovery of *Pn*fucA markedly broadens the array of tools available for glycoengineering applications.

**Table 1:** Kinetic Parameters of Different GH29A α-l-Fucosidases with *p*NP-Fucose as the Substrate

organism	*k*_cat_ (s^–1^)	*K*_m_ (mM)	*k*_cat_/*K*_m_ (s^–1^ mM^–1^)	sequence identity^*^ (%)	refs
*Thermotoga maritima*	5.4 ± 0.2	0.03 ± 0.00	158,8	21,7	(32)
*L. casei* AlfC	1.38 ± 0.02	0.70 ± 0.03	2,0	18,6	(33)
*Bacteroides fragilis* BfFucH	183,8	0,44	420,6	53,1	(29)
*Bacteroides thetaiotaomicron* BT-2970	1,3	1,5	0,87	22,3	(37)
*Elizabethkingia anophelis* cFase	0.14	0.60	0.23	23,1	(38)
*P. nigrescens* Δ20*Pn*fucA	66.5 ± 2	0.56 ± 0.05	118,8		this study
*P. nigrescens* MBP-*Pn* fucA	35.4 ± 0.64	0.25 ± 0.02	141,6		this study
*L. casei* MBP-AlfC	9.7 ± 0.28	0.62 ± 0.06	15,6		this study

## Results and Discussion

### Bioinformatic Analysis of the *Pn*fucA Sequence

*Pn*fucA belongs to the GH29A subfamily, but it exhibited relatively low sequence identity when compared with other characterized GH29A α-l-fucosidases (Figure S1). Specifically, it shares only 18.6% sequence identity with *L. casei* α-l-fucosidases (AlfC),^31^ 21.7% with *Thermotoga maritina* α-l-fucosidases (TM aFuc),^32^ and 53.1% identity to the closest α-l-fucosidase homologue the *B. fragilisBf*FucH.^29^ The conserved catalytic nucleophile D223 in *Pn*fucA has been putatively identified and is aligned to both nucleophiles D224 in TM aFuc and D200 in AlfC (Figure S2).^32,33^ To investigate the possible acid/base residues based on a structural homology study with other characterized CH29A α-l-fucosidases,^33−35^ we generated a theoretical three-dimensional (3D) structural model of *Pn*fucA using AlphaFold 2 (Figure S3A,B). The projected structure showed a classic GH29 (β/α)_8_ barrel structure connected to a *C*-terminal β-sandwich domain with a high degree of reliability, as indicated by a high per-residue confidence metric (pLDDT; predicted local distance difference test) of over 90. The *N*-terminal region of the model displayed a very low confidence score (pLDDT < 50). This region comprises signal peptide residues 1–20, and, due to poor prediction quality, this region was removed from the model. A structure superposition analysis verified that the catalytic amino acid residues in *Pn*fucA are E273 (general acid/base) and D242 (nucleophile). These residues are aligned similarly to those in TM aFuc and human α-l-fucosidase (FucA1) (Figure S3C,D), but they are different from those in AlfC (Figure S3E). It is also important to note that GH29 enzymes function as retaining hydrolases, using the classic Koshland double-displacement catalytic reaction.^36^

### Heterologous Expression of α-l-Fucosidases

Recognizing *Pn*fucA as a potential GH29A α-l-fucosidase, we embarked on producing recombinant *Pn*fucA in *Escherichia coli* cells. As a positive control, we also produced recombinant *L. casei* fucosidase AlfC. Our initial attempt using the pET-22b+ vector resulted in insoluble proteins. To improve protein solubility, we introduced the *N*-terminal Maltose binding protein (MBP) tag into the pMAL-c4X vector which effectively enabled the expression of both *Pn*fucA and AlfC in a soluble form (Figure S4). Although functionally the MBP-*Pn*fucA preparation showed remarkable catalytic efficiency (as shown in Figure S5), the low yields (<30 μg/L) of MBP-fusion *Pn*fucA with poor purity (Figure S4) were a significant challenge and constraint for wider and more versatile applications. Following promising preliminary investigations, we adopted pET-16b with 20 amino acids removed from the *N*-terminal region, and this led to the successful production of *Pn*fucA with an apparent molecular weight of ∼52 kDa (Figure S4), referred to as △20*Pn*fucA. After affinity purification, this approach gave a 1000-fold increase in yield to over 30 mg/L with outstanding purity. This substantial yield of △20*Pn*fucA enabled us to proceed with the enzyme’s biochemical characterization.

### Biochemical Characterization of Heterologous Expressed *Pn*fucA Enzyme

**Figure 1:**
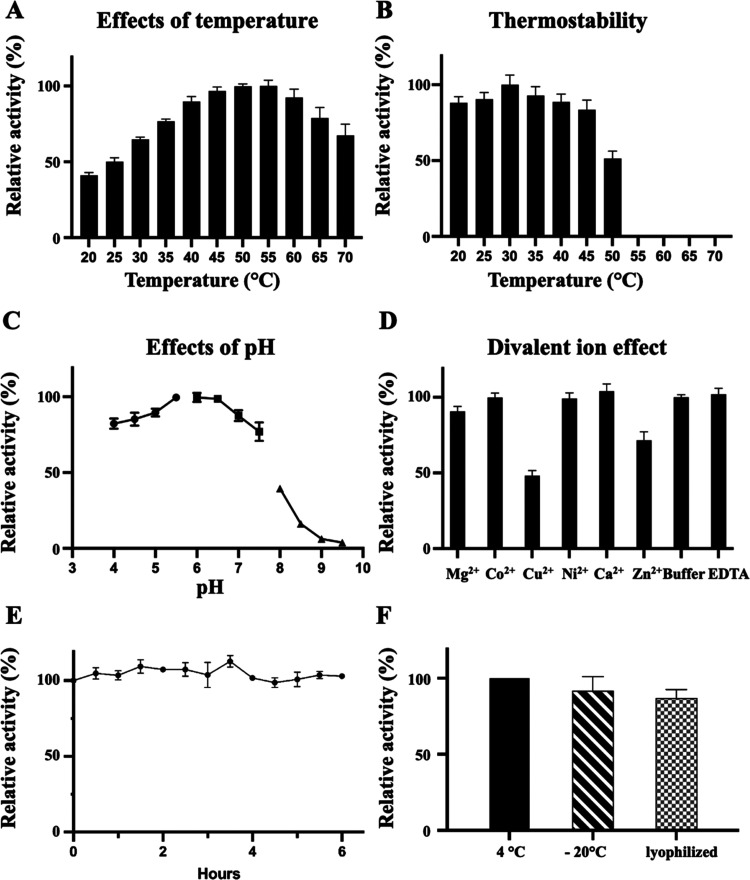
Effects of temperature, pH, and divalent ions on the catalytic activity of △20*Pn*fucA. The optimal reaction temperature was determined by incubating the enzyme at different temperatures (A). Thermostability was assayed by incubating the enzyme at different temperatures for 10 min before performing the enzymatic activity assay (B). The optimal pH was determined using sodium acetate (pH 4 to pH 5.5, circle), sodium phosphate (pH 6 to pH 7.5, square), and Tris (pH 8 to 9.5, triangle) buffers (C). The effects of divalent ions on enzyme activity were determined using 50 mM sodium phosphate buffer (pH 7) containing 5 mM of either divalent ions or EDTA (D). The effects of incubation time were evaluated by incubating the enzyme at 37 °C in sodium phosphate buffer (pH 7) for up to 6 h (E). The effects of storing the enzyme in 50 mM sodium phosphate buffer at 4 °C and −20 °C, or as a freeze-dried powder at 4 °C were evaluated after storage for 28 days. Relative activity values were determined compared with the highest activity values (measured at 55 °C in (A), at 30 °C in (B), and at pH 6 in (C)), with the buffer-only sample (D), with the starting time point at 0 h (E), and with freshly purified enzyme on day 1 (F). The experiments were performed in triplicate, and error bars represent the standard deviation.

In assessing the biochemical characteristics of recombinant △20*Pn*fucA, synthetic substrate *p*-nitrophenyl-α-l-fucopyranoside (*p*NP-Fuc) was used as the substrate of choice. On enzymatic hydrolysis, *p*NP-Fuc undergoes cleavage to release *p*-nitrophenyl (*p*NP). The optimal activity of △20*Pn*fucA was at 50–55 °C, and the enzyme retained over 80% of its activity at temperatures below 45 °C (Figure 1A,B). The optimal performance of the enzyme was at the slightly acidic pHs of 5.5–6.5, and the presence of copper and zinc ions was detrimental to its activity (Figure 1C,D). Full activity was maintained even after incubation (at pH 7) for 6 h at 37 °C (Figure 1E). To further assess its stability, we stored the enzyme at −20 °C and after freeze-drying. Remarkably after 28 days of storage, over 92 ± 9 and 87 ± 6% of its activity was retained at −20 °C and in the freeze-dried state, respectively (Figure 1F).

The kinetics parameters *k*_cat_ and *K*_m_ for △20*Pn*fucA were calculated at 66.5 ± 2 s^–1^ and 0.56 ± 0.05 mM, respectively. The △20*Pn*fucA exhibited a significantly higher *V*_max_ (77.2 ± 0.1 U/mg) compared with those of the MBP-fusion *Pn*fucA (22.2 ± 0.4 U/mg) and MBP-fusion AlfC (7.0 ± 0.2 U/mg), as shown in Figure S5. This observation indicated that fusion with MBP negatively affects *Pn*fucA activity. Although *p*NP-Fuc is a commonly used substrate for the enzymatic characterization of GH29A α-l-fucosidases, GH29B enzymes do not hydrolyze this synthetic substrate.^37^ Furthermore, hydrolysis of *p*NP-Fuc does not elucidate the enzyme’s regiospecificity for α(1,2)-, (1,3)-, (1,4)-, and (1,6)-linked substrates. For instance, both *B. thetaiotaomicron* BT-2970 and *Elizabethkingia meningoseptica* cFase are active with *p*NP-Fuc, but are inactive with the Fucα(1,6)GlcNAc disaccharide, as indicated in Tables 1 and S1.^29,31−33,37−42^ Therefore, further investigations using natural disaccharide substrates are necessary to make a meaningful kinetic comparison among the characterized α-l-fucosidases.

### Analysis of the Substrate Specificity of *Pn*fucA

The substrate specificity of △20*Pn*fucA was determined by incubating the enzyme in a range of synthetic glycans (di-, tri-, and oligosaccharides) containing fucose residue(s) and the reducing end C-5 amino linker (Table 2 and Figure S6). The presence of the C-5 amino linker does not alter the structure of glycan substrates and is employed for subsequent conjugation with Cy5Mono NHS Ester. After reacting for 16 h, the products were analyzed using both high-performance liquid chromatography (HPLC) and liquid chromatography–mass spectrometry (LC-MS) (Figures S7 and S8). With the substrates having core fucosylated disaccharides, *Pn*fucA showed the greatest ability to liberate fucose from 3FN-C5, 4FN-C5, and 6FN-C5. The HPLC analysis showed that the substrates were completely hydrolyzed. Furthermore, the LC-MS analysis showed that the extent of fucose removal surpassed 95% for both 3FN-C5 and 6FN-C5, highlighting the enzyme’s exceptional effectiveness in cleaving this substrate category (Table 2). Interestingly, we found no evidence of hydrolytic activity against the C-5 linked Lewis antigen x (Le^x^), Lewis antigen a (Le^a^), fucosylated GM3 ganglioside 3 (FGM3), Globo A, and subterminal α(1,4)-fucosylated or α(1,6)-fucosylated disialyl-*N*-glycans (Table 2). This clearly demonstrated that *Pn*fucA has no activity on subterminal fucosylated oligosaccharides.

**Table 2:**
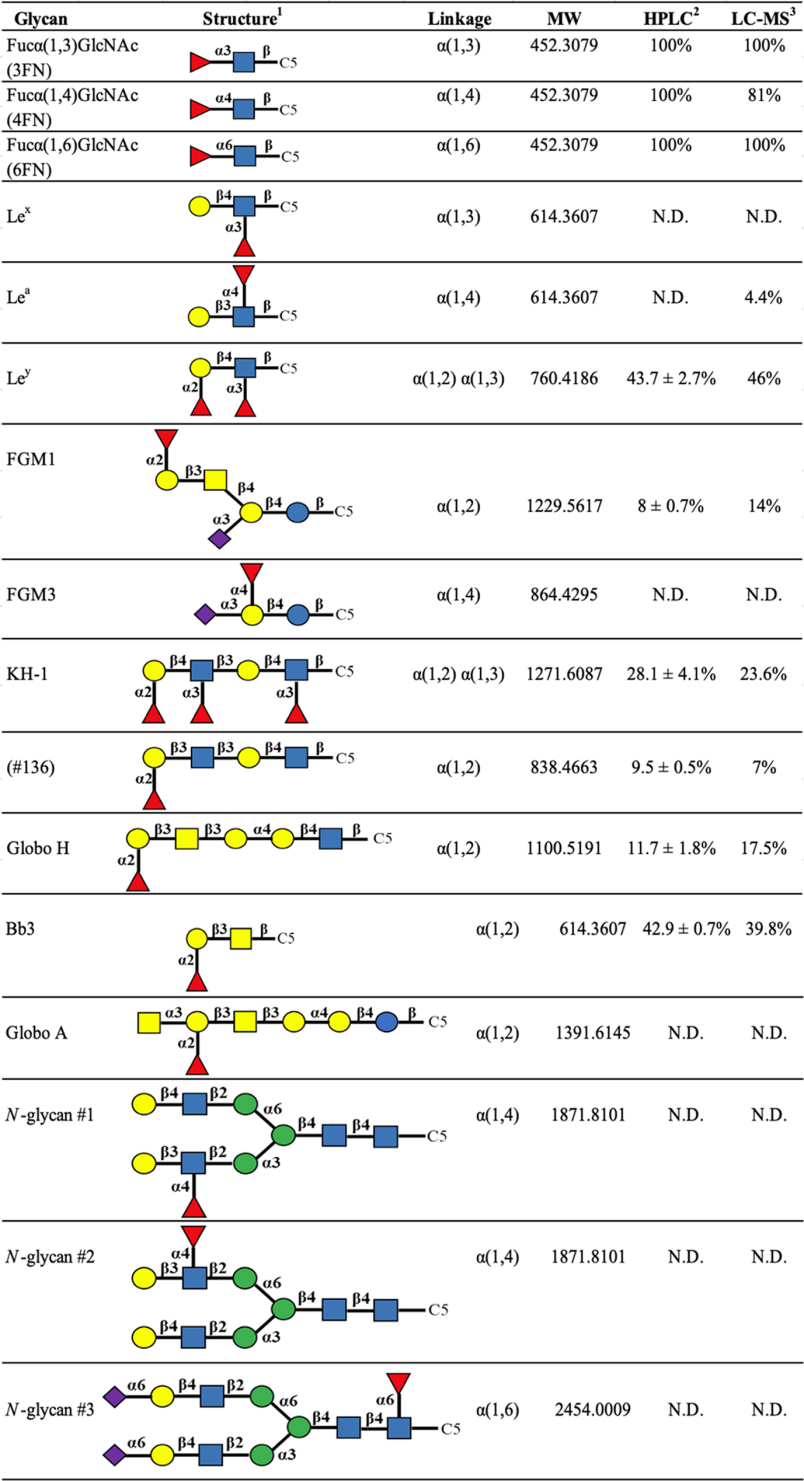
Substrate Specificity of △20*Pn*fucA

1 Blue circle: glucose; blue square: *N*-acetylglucosamine; yellow circle: galactose; yellow square: *N*-acetylgalactosamine; red triangle: fucose; violet diamond: sialic acid.

2 Hydrolysis rate determined by HPLC analysis. The values indicated mean ± standard deviation.

3 Hydrolysis rate determined by LC-MS analysis.

There was moderate hydrolytic activity detected on C-5 linked fucosylated GM1 ganglioside 1 (FGM1), synthetic oligosaccharide #136 (pentasaccharide with a terminal α(1,2)-linked fucose), Globo H, and Bb3 glycans. Nevertheless, only 8–42.9% of terminal fucose was released from these substrates with higher degrees of polymerization (DPs). This implies a preference for core FN. With di- and trifucosylated substrates, such as Lewis antigen y (Le^y^) and antigen KH-1, hydrolysis removed a single fucose residue (−146 Da), demonstrating the enzyme’s ability to target and remove terminal fucose residues. The substrate preferences of some GH29A α-L-fucosidases are summarized in Table S1. As with the recently identified AlfC, △20*Pn*fucA showed broad linkage specificity with a marked preference for disaccharides substrates (Table 2), whereas BT-2970 hydrolyzed only the subterminal fucosylated substrates Le^x^ and Le^y^ but not core FN.

### Defucosylation of *N*-Linked Glycoprotein Using *Pn*fucA

Prior to conducting the defucosylation reactions on the model *N*-linked glycoprotein mAb adalimumab (Humira), we analyzed the structural profiles of *N*-glycans on Asn 297 in the Fc segment of adalimumab. Consistent with the literature,^43^ the LC-ESI-MS analysis revealed that all of the *N*-glycans were fucosylated, with a distribution of 2.4% mono-*N*-acetylglucosaminylated (G0F–N), 84.7% agalactosylated (G0F), and 12.9% monogalactosylated (G1F) complex-type *N*-glycans (Table S2).

The extent of defucosylation of adalimumab by △20*Pn*fuc was monitored by using Western blot analysis with the fucose-specific *Aleuria aurantia* lectin (AAL). The presence of α(1,6)-linked Fuc*p* was confirmed by positive AAL staining in both untreated adalimumab and Endo-S2-treated adalimumab. Furthermore, when adalimumab was treated only with △20*Pn*fucA, strong AAL staining was observed, indicating the enzyme failed to remove the core fucose in extended *N*-glycans. This is also consistent with the failure of △20*Pn*fucA to hydrolyze the fucose residues on C-5 linked α(1,6)-core fucosylated sialyl-comple-type *N*-glycan (*N*-glycan #3, Table 2 and Figure S8P).

**Figure 2:**
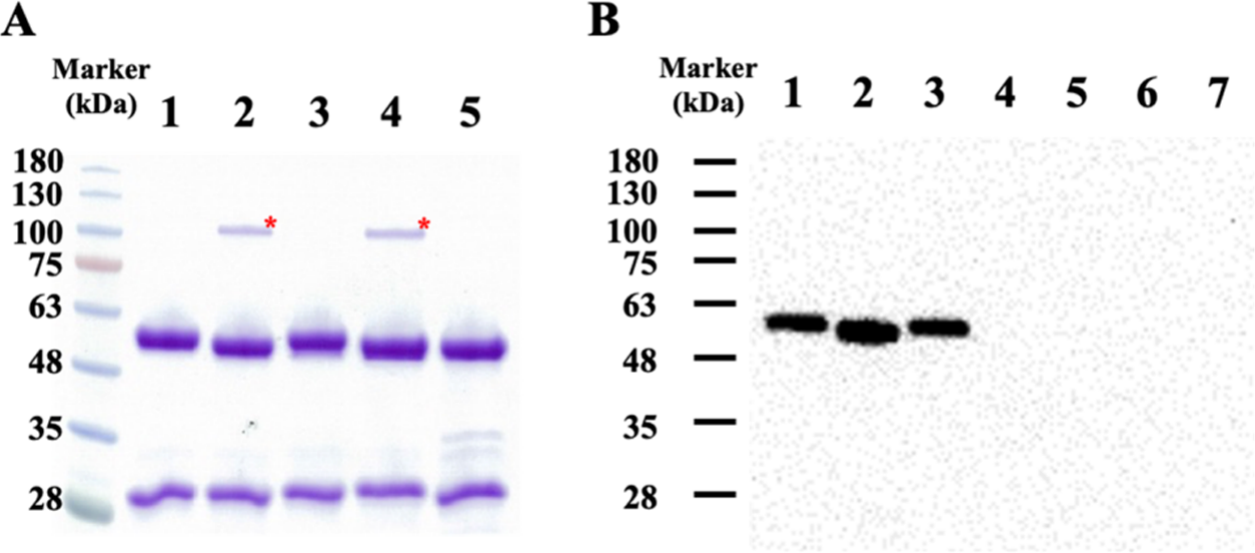
Removal of the core α(1–6) fucose of adalimumab by △20*Pn*fucA. Hydrolysis products were loaded for SDS-PAGE (A) and for lectin blotting (B). Untreated adalimumab (lane 1), Endo-S2 deglycosylation (lane 2), △20*Pn*fucA defucosylation (lane 3), combined Endo-S2 and △20*Pn*fucA treatment (lane 4), PNGase F deglycosylation (lane 5), fetuin (afucosylated *N*-linked glycoprotein; lane 6), and BSA (lane 7). Endo-S2 is indicated by *.

**Figure 3:**
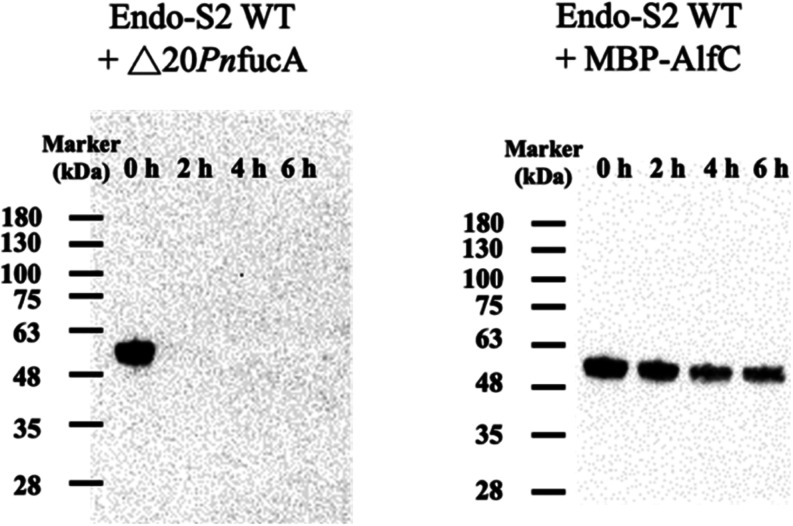
Defucosylation of adalimumab using a combination of wild-type Endo-S2 and an α-l-fucosidase. Adalimumab (50 μg) was treated with Endo-S2 (5 μg) combined with either △20*Pn*fucA (5 μg) or MBP-AlfC (5 μg). 2 μg of protein was loaded for lectin blotting analysis. Samples were collected at 0, 2, 4, and 6 h post hydrolysis.

Following the treatment of adalimumab with Endo-S2 to give adalimumab containing a core 6FN disaccharide (Hum_Fucα(1,6)-GlcNAc_), defucosylation was carried out with △20*Pn*fucA, leading to the complete removal of core fucose from Hum_Fucα(1,6)-GlcNAc_, with a specific activity of 9.2 ± 1.3 μg per μg of △20*Pn*fucA per hour. This finding suggests that *Pn*fucA selectively targets terminal fucose residues, such as those in core FN (Figure 2). A comparative study was undertaken using MBP-fusion α-l-fucosidases. Using △20*Pn*fucA, defucosylation was fast, with MBP-*Pn*fucA achieving complete defucosylation within 2 h, whereas MBP-AlfC showed a slower defucosylation rate, with residual core-α(1,6) fucose still present after 6 h (Figure 3). Interestingly, sequence alignment analysis showed a notable difference between the two enzymes. The targeting of Fucα(1,6)GlcNAc by AlfC was attributed to the GlcNAc residue within the hydrophobic pocket formed by Trp 40, Ala 154, and Trp 158. Specifically, the Ala 154 and the Trp 158 are situated within a loop consisting of 19 amino acids (ranging from Gly 145 to Asp 163), whereas *Pn*fucA has a much shorter loop with only 10 amino acids (from Ile 181 to Arg 190), as shown in Figure S2. The absence of the homologous hydrophobic pocket in *Pn*fucA may have an impact on the substrate binding of these two enzymes, as shown in Figure S9.

### One-Pot Two-Step Fc Domain Glycoengineering Using *Pn*fucA

**Figure 4:**
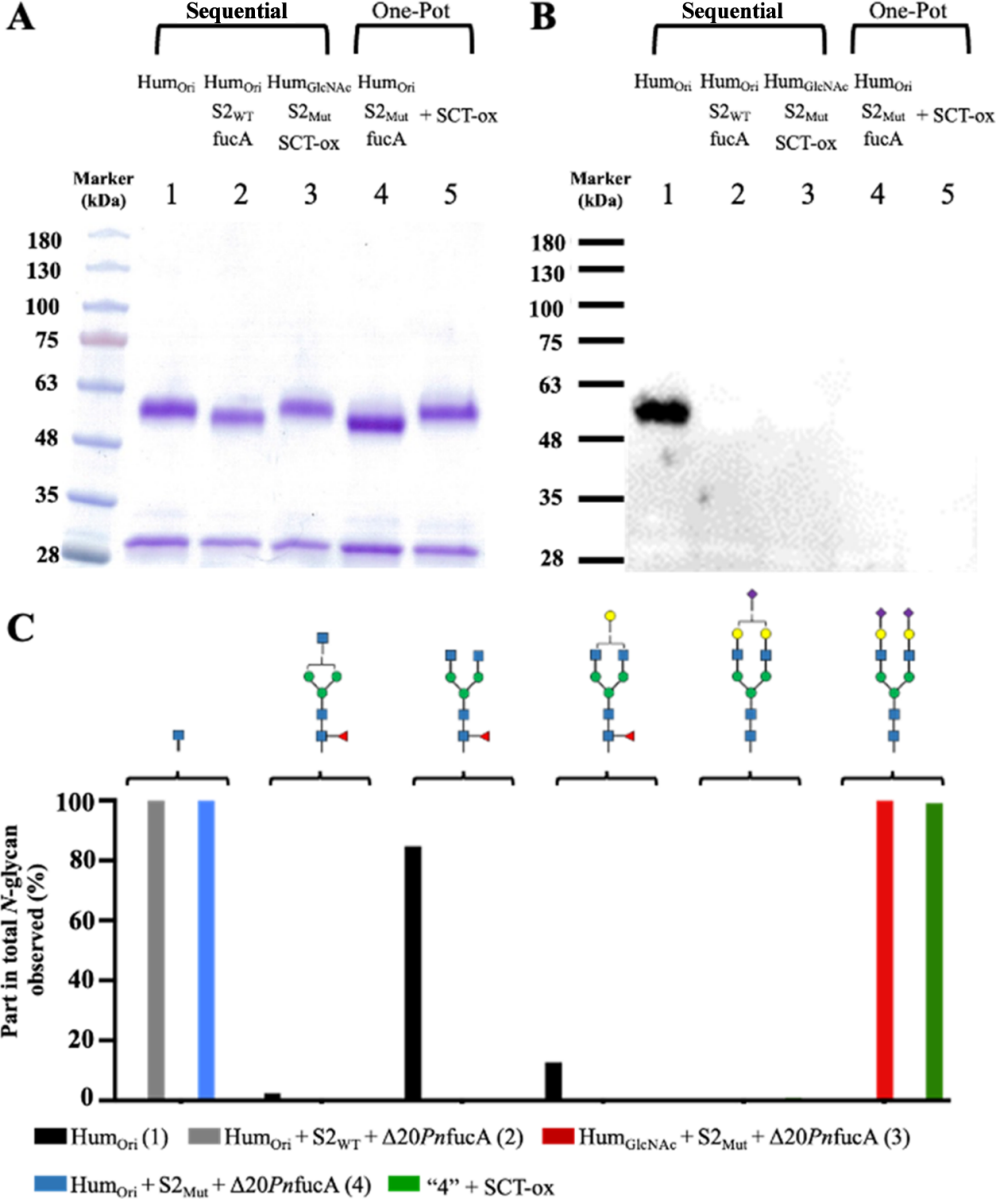
Transglycosylation of afucosylated adalimumab with sequential deglycosylation/transglycosylation reactions, or with a one-pot two-step reaction. Posthydrolysis and transglycosylation samples were analyzed using SDA-PAGE (A) and lectin blotting (B). Lane 1: untreated adalimumab (Hum_Ori_); Lane 2: combined wild-type Endo-S2 (S2_WT_) and △20*Pn*fucA (fucA) treatment; Lane 3: transglycosylation of afucosylated adalimumab (Hum_GlcNAc_) with Endo-S2 mutant D184 M (S2_Mut_) and SCT-oxazoline (SCT-ox); Lane 4: combined Endo-S2 mutant D184 M (S2_Mut_) and △20*Pn*fucA (fucA) treatment; Lane 5: transglycosylation after the 3 h treatment with the Endo-S2 mutant D184 M and △20*Pn*fucA, followed by the immediate addition of SCT *N*-glycan oxazoline (+SCT-ox). Composition of the Fc *N*-glycans (C). Corresponding mass values for each glycosylated peptide from thermolysin-digested adalimumab are detailed in Supporting Table 2. See Table 1 for the identities of the symbols representing glycan structures.

The currently used method for glycoengineering mAbs to enhance effector functions involves a three-step process using three enzymes: an endo-β-*N*-acetylglucosaminidase and its mutant glycosynthase, together with a GH29 α-l-fucosidase (Scheme 1). Using adalimumab (Humira) as an example, to produce a uniform α(2,6)-sialylated biantennary complex-type (SCT) *N*-glycan, we initiated the process with Endo-S2, which removed heterogeneous Fc *N*-glycans. Subsequently, △20*Pn*fucA was used to remove the core-α(1,6) fucose residue, resulting in adalimumab with the first GlcNAc bridgehead as the acceptor (Hum_GlcNAc_) (Figure 4A–C, lane 1). After Hum_GlcNAc_ was purified (Figure S10), transglycosylation was carried out using the Endo-S2 mutant D184 M, with SCT-oxazoline (SCT-ox) as the sugar donor, yielding glycoengineered adalimumab with a uniform SCT *N*-glycan (Hum_SCT_) (Figure 4A–C, lane 3) in quantitative yield within 1 h. In our study, we found that Endo-S2 mutant D184 M can also perform hydrolysis effectively on Fc *N*-glycans of adalimumab, achieving a rate of 54.6 ± 13.7 μg per μg of enzyme per hour, despite it being previously reported that this mutant enzyme showed only very low hydrolytic activity (about 1.4% compared with the wild-type enzyme)^27^ (Figure 4A–C, lane 4). Additionally, the transglycosylation rate was found to be 74.5 ± 2.9 μg of adalimumab per μg of Endo-S2 mutant D184 M per hour.

We then evaluated and optimized the SCT-ox concentration for the transglycosylation reaction (Figure S11). After a 1 h reaction, the conversion to the transglycosylated product Hum_SCT_ was 100, 71, and 6% in the presence of 10, 1, and 0.1 mM SCT-ox, respectively. The proportion of Hum_SCT_ decreased from the second hour and dropped abruptly after the fourth hour to 8% for 1 mM SCT-ox and 43% for 10 mM SCT-ox. In contrast, for 0.1 mM SCT-ox, it remained around 7% between the first and fourth hours and fell to 2% at the sixth hour. Based on these results, we propose a one-pot two-step reaction to generate homogeneous Hum_SCT_ within 4 h, consisting of 3 h of deglycosylation followed by 1 h of transglycosylation. In brief, for the first step, the antibody adalimumab was defucosylated in the presence of △20*Pn*fucA and the Endo-S2 mutant. The reaction was successfully completed within 3 h to afford Hum_GlcNAc_ with a quantitative yield, as shown in lane 4 in Figure 4A–C. Subsequently, in the same reaction vessel, we introduced 10 mM SCT-ox and allowed it to react for 1 h, resulting in the transglycosylated product Hum_SCT_ with a 100% yield (Figure 4A–C, lane 5).

**Figure 5:**
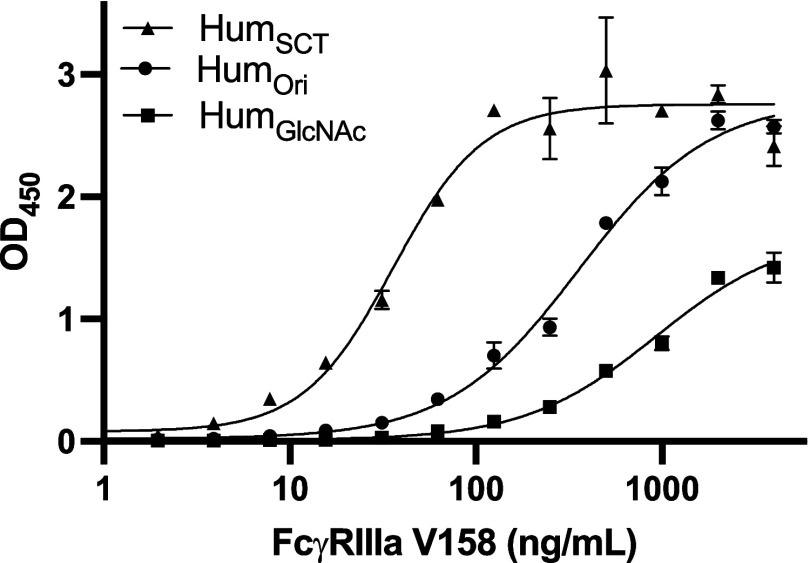
ELISA dose–response curve for glycoengineered adalimumab with the Fcγ receptor IIIa. The FcγRIIIa V158 variant was coated on the ELISA plate with a concentration of 0.5 μg/mL. Adalimumab glycoforms Hum_Ori_, Hum_SCT_, and Hum_GlcNAc_ were obtained from the one-pot two-step reaction and were purified by protein A chromatography. The Goat (Fab′)_2_ antihuman IgG Fc conjugated with the horseradish peroxidase was used as a detector antibody. The experiments were performed in duplicate, and error bars represent the standard deviation.

In a previous study, a uniform and defucosylated rituximab (Rituxan) was produced^9^ through a series of three distinct enzymatic reactions using three different enzymes: wild-type Endo-S2, AlfC, and the mutant Endo-S2 D184 M. Following each enzymatic reaction, purification was essential and was accomplished by protein A chromatography.^9,27^ However, in our study, we used △20*Pn*fucA with only the mutant Endo-S2 D184 M in a one-pot method that is an effective and straightforward strategy for glycoengineering. This approach streamlined the transformation of a heavily fucosylated monoclonal antibody, synthesized in CHO cells, into a uniformly defucosylated glycoform within 4 h, eliminating the need for extra purification steps. With Hum_GlcNAc_ and Hum_SCT_ in hand, obtained from the one-pot reaction (Figure 5), we found that the binding affinities of Hum_SCT_ to FcγRIIIa V158 are highest (EC_50_ = 35.9 ng/mL) compared to Hum_Ori_ (EC_50_ = 358.4 ng/mL) and Hum_GlcNAc_ (EC_50_ = 928.6 ng/mL). This also translated to enhanced ADCC activity, showcasing the potential applications of an improved antibody glycoengineering strategy.

## Conclusions

Several α-l-fucosidases have been investigated for their diverse hydrolytic activities, but only a limited number have demonstrated high efficiency in cleaving the α(1,6)-linked fucose. The GH29A α-l-fucosidase *Pn*fucA was shown to have potential in the remodeling of the Fc *N*-glycans of monoclonal antibodies. This enzyme efficiently hydrolyzed α(1,3)-, (1,4)- and (1,6)-linked core fucose as in fucosylated disaccharides and showed moderate activity on terminal linked α(1,2)-linked fucose in fucosylated oligosaccharides. Kinetic analysis of this enzyme, including key parameters such as *K*_m_ and *k*_cat_, has provided valuable insights. In the glycoengineering of adalimumab, △20*Pn*fucA showed exceptional hydrolytic efficiency, particularly in targeting α(1,6)-linked core fucose, achieving a quantitative yield. This noteworthy performance facilitated the seamless integration of biantennary sialylglycans in a one-pot two-step approach, resulting in adalimumab with a homogeneous glycoform. This capability allows researchers to selectively modify core fucosylation, thereby optimizing Fc effector functions for enhanced therapeutic outcomes. The strategic use of △20*Pn*fucA in glycoengineering opens new avenues for tailoring the glycosylation profile of the Fc glycan in mAbs and other Fc-fusion proteins, offering improved precision and control over glycan structures in therapeutic intervention.

## Methods

### Chemicals and Plasmids

Analytical- or reagent-grade chemicals were used unless specified and were purchased from Fisher Scientific (U.K.) and Sigma-Aldrich (St Louis). The synthesized and codon-optimized DNA sequences encoding the α-l-fucosidases of *P. nigrescens* (*Pn*fucA) and *L. casei* (AlfC), as well as the expression vectors (pET-16b, pET-22b+ and pMAL-c4X) were from GenScript (US).

### Evolutionary Study of α-l-Fucosidases

Protein sequences of α-l-fucosidases were selected from databases including the National Center for Biotechnology Information (NCBI, https://www.ncbi.nlm.nih.gov/), Carbohydrate-Active enZYmes (CAZy, http://www.cazy.org/),^28^ and the RCSB Protein Data Bank (PDB, https://www.rcsb.org/).^44^ Multiple sequence alignment was conducted with Clustal Omega,^45^ and the phylogenetic tree was built using the Neighbor-Joining method with MEGA11.^46^ Signal peptides were predicted with SignalP version 6.0.^47^ The 3D structure of *Pn*fucA was predicted using AlphaFold 2^48^ and visualized with the PyMol Molecular Graphics System (Version 1.2r3pre, Schrödinger, LLC).

### Substrate Specificity Experiment

The fucosylated oligosaccharide substrates were synthesized as previously described.^49,50^ The reaction was performed in a final volume of 50 μL, containing 1 μg of purified Δ20*Pn*fucA and 2 mM of the fucosylated substrate with a C-5 amino linker (Figure S6), in 50 mM sodium phosphate buffer (pH 7), and incubated at 37 °C for 16 h. After the hydrolysis, the reaction products were purified using a Bond Elut Carbon cartridge (Agilent) that contained 50 mg of carbon in a 1 mL column. The carbon was activated with 3 mL of 80% acetonitrile and then washed with 3 mL of Milli-Q (MQ) water. A 200 μL aliquot diluted with MQ water was injected onto the column, which was then washed with 3 mL of MQ water and eluted with 500 μL of 50% acetonitrile. The eluted fraction was freeze-dried overnight.

The hydrolysis products of the fucosylated substrates were analyzed by high-performance liquid chromatography (HPLC) and mass spectrometry (MS) methods. Before HPLC analysis, dried samples were dissolved in 50 mM sodium phosphate buffer (pH 8)(19 μL), labeled with 1 μL of 12 mM Cy5Mono *N*-hydroxysuccinimide (NHS) ester (GE Amersham), and incubated at RT overnight. Analytical HPLC was carried out with an XBridge Glycan Ethylene Bridged Hybrid (BEH) amide Column (130 Å, 3.5 μm, 2.1 mm × 150 mm, Waters) on a Waters e2695 Separation Module, paired with a Waters 2998 photodiode array detector (PDA). Cy5-labeled glycans were detected with an absorption wavelength of 649 nm. The mobile phase consisted of solvent A (100 mM ammonium formate, pH 4.5) and solvent B (100% acetonitrile), and the separation was conducted with gradient elution from 90 to 45% of solvent B over 40 min (flow rate, 0.25 mL/min). High-resolution MS was performed on an LTQ Orbitrap XL ETD (Electron Transfer Dissociation) mass spectrometer (Thermo Fisher Scientific) equipped for UPLC (ultraperformance liquid chromatography) (Waters Acquity) with an XBridge BEH C18 Column (130 Å, 3.5 μm, 1 mm × 150 mm, Waters).

### Deglycosylation and Transglycosylation of Adalimumab (Humira)

*Streptococcus pyogenes* endo-β-*N*-acetylglucosaminidase S2 (Endo-S2) wild-type and mutant D184 M enzymes were produced in-house. The deglycosylation reaction was performed with 50 μg of adalimumab and 5 μg of Endo-S2 in 50 mM sodium phosphate buffer (pH 7) at 37 °C for 3 h. The defucosylation reaction was performed with 50 μg of Endo-S2-treated adalimumab and 5 μg of α-l-fucosidase in 50 mM sodium phosphate buffer (pH 7) at 37 °C for 3 h. Transglycosylation was conducted at 37 °C for 1 h with 50 μg of deglycosylated protein acceptor, 5 μg of Endo-S2 D184 M mutant, and 10 mM sialyl complex-type *N*-glycan oxazoline (SCT-ox, produced in house) in 50 mM Tris-HCl buffer (pH 7.6), complemented with 50 mM NaCl and 1 mM CaCl_2_. Enzyme-treated adalimumab was purified using a Protein A Spin Antibody Purification Kit (BioVision). The purified adalimumab was then digested by thermolysin, and the Fc domain *N*-glycan was determined using LC-ESI-MS on an Orbitrap Fusion MS (Thermo Scientific) equipped with an Easy-nLC 1200 system and an Easy-Spray source. Samples were injected into a C18 Easy column (0.075 mm × 150 mm, ID 3 μm).

### Lectin Blotting

The presence of α(1,6)-linked core fucose in glycoproteins was detected by using Western blotting with biotinylated *A. aurantia* lectin (AAL) (Vector Laboratories). After electrophoretic separation, sample proteins were transferred onto the Immun-Blot PVDF membrane (Bio-Rad) using the Criterion blotter system (Bio-Rad). The membrane was then incubated at 4 °C overnight in a blocking solution composed of PBS (OmicsBio, Taiwan) supplemented with 0.05% Tween-20 (PBS-T) and 3% BSA. The blotting process started with incubation in 10 mL of blocking buffer containing 2 μg/mL AAL at RT for 1 h, followed by washing three times for 30 min each with PBS-T. Subsequently, the membrane was incubated in 10 mL of blocking buffer supplemented with 10 μL of Avidin-horseradish peroxidase (HRP) conjugate (Invitrogen) at RT for 1 h. After washing, the fucosylated *N*-linked glycans were visualized upon the addition of Western Lightning Plus Chemiluminescent Substrate (PerkinElmer) and images were captured by using the iBright 1500 Imaging System (Thermo Fisher Scientific).

## Supporting Information Available

Additional experimental details, materials and methods, and HPLC and MS data (PDF)
