# Supplementary material for: A Robust α-l-Fucosidase from Prevotella nigrescens for Glycoengineering Therapeutic Antibodies
Source: ACS Chem Biol. 2024 Jun 24;19(7):1515–24. doi: 10.1021/acschembio.4c00196 (PMC11267573; doi:10.1021/acschembio.4c00196)
Supplement: Supplementary file 1 — cb4c00196_si_001.pdf [file cb4c00196_si_001.pdf]

Supporting Information

A Robust α-L-Fucosidase from *Prevotella nigrescens* for Glycoengineering Therapeutic Antibodies

Mu-Rong Kao[†,‡,±], Tzu-Hsuan Ma[†,±], Hsiang-Yu Chou[†], Shu-Chieh Chang[±], Lin-Chen Cheng[†], Kuo-Shiang Liao[‡], Jiun-Jie Shie[¶], Philip J. Harris[§], Chi-Huey Wong[‡,#] and Yves S. Y. Hsieh[†,‡,±*]

[†]School of Pharmacy, College of Pharmacy, Taipei Medical University, No. 250 Wuxing Street, Taipei, 11031, Taiwan

[‡]Genomics Research Center, Academia Sinica, No. 128 Academia Road, Section 2, Nankang District, Taipei, 115201, Taiwan

[±]Division of Glycoscience, Department of Chemistry, School of Engineering Sciences in Chemistry, Biotechnology and Health, Royal Institute of Technology (KTH), AlbaNova University Centre, Stockholm, SE-10691, Sweden

[¶]Institute of Chemistry, Academia Sinica, No. 128 Academia Road, Section 2, Nankang District, Taipei, 115201, Taiwan

[§]School of Biological Sciences, The University of Auckland, Auckland Mail Centre, Private Bag 92019, Auckland 1142, New Zealand

[#]Department of Chemistry, The Scripps Research Institute, 10550 North Torrey Pines Road, La Jolla, California 92037, United States

[*]To whom correspondence should be addressed: email. yvhsieh@kth.se

Material and Methods

Expression and purification of recombinant α-L-fucosidases

The coding sequence of α-L-fucosidase was amplified using Polymerase Chain Reaction (PCR) with the Fusion High-Fidelity DNA Polymerase (Thermo Fisher Scientific Baltics, Lithuania), following the manufacturer's recommendations. It was then cloned into the expression vector after linearization with appropriate restriction enzymes, which were purchased from New England BioLabs (NEB, UK). The resulting expression vectors were transformed into *E. coli* DH5α strain (Yeastern Biotech, Taiwan) for plasmid preservation and subsequently into the *E. coli* BL21 strain (Yeastern Biotech, Taiwan) for recombinant protein expression. Transformants were selected from Luria-Bertani (LB) medium agar plates supplemented with 100 μg/mL ampicillin.

For recombinant enzyme production, a single bacterial colony was inoculated into LB medium supplemented with ampicillin and cultured at 37°C overnight. The next day, fresh LB medium was inoculated with the overnight culture to get a starting optical density (OD_{600}) of 0.1 and was cultured at 37°C until the OD_{600} reached between 0.6 and 0.8. The culture was then induced with 0.5 mM isopropyl 1-thio-β-D-galactopyranoside (IPTG, AK Scientific, US) and incubation continued at 16°C for an additional 16 hours. Cells were collected by centrifugation at 6000 *g* for 5 min. The resulting cell pellet was resuspended in binding buffer for protein purification, and cell lysis was performed with the One Shot Cell Disruptor equipment (Constant Systems, UK).

The purification of recombinant α-L-fucosidases was carried out by affinity chromatography with an AKTA Purifier FPLC System (GE Healthcare, Sweden). His-tagged recombinant enzymes were purified with a HisTrap FF column (Cytiva, Sweden)

and a phosphate binding buffer (20 mM sodium phosphate, 500 mM NaCl and 20 mM imidazole, pH 7.4). After the sample injection, the column was washed with 15 column volumes (CV) of binding buffer and subsequently eluted with 20 CV of elution buffer (20 mM sodium phosphate, 500 mM NaCl and 500 mM imidazole, pH 7.4) with a linear gradient from 0 to 100%. For MBP-fusionned proteins, purification was performed with an MBPTrap HP column (Cytiva, Sweden). The binding buffer contained 20 mM Tris-HCl, 200 mM NaCl and 1 mM Ethylenediaminetetraacetic acid (EDTA) at pH 7.4, and the elution buffer was prepared with the same buffer supplemented with 10 mM maltose. The purity of proteins in the elution fractions were verified by SDS-PAGE analysis using the TGX Stain-Free FastCast Acrylamide Kit (Bio-Rad, US). Fractions showing a strong band of the target protein were gathered and concentrated using an Amicon Ultra Centrifugal Filter Unit (Merck, Germany). Following buffer exchange with 50 mM sodium phosphate buffer (pH 6.5), the protein concentration of the purified enzyme was determined by the Bradford assay[1] using Bio-Rad Protein Assay (Bio-Rad, US), with bovine serum albumin (BSA) serving as the standard.

Enzymatic activity experiment

The enzyme activity of α-L-fucosidases was determined with 0.03 ug of purified Δ20*Pn*fucA and 2 mM of the artificial substrate *p*-nitrophenyl-α-L-fucopyranoside (*p*NP-fucose, Carbosynth, UK). The reaction was carried out in 50 mM sodium phosphate buffer (pH 6.5) for 5 min at 37°C. The hydrolysis product, *p*NP, was detected by spectrophotometry using a BioDrop Duo+ (Biochrom, UK) at OD_{405}. Its concentration was quantified using a standard curve generated from serially diluted *p*NP solutions, ranging

from 0.016 to 0.5 mM. One enzyme unit (U) was defined as 1 μmol of *p*NP released per minute.

The effects of pH were determined using 50 mM sodium acetate (pH 4 to 5.5), sodium phosphate (pH 6 to 7.5) and Tris (pH 8 to 9.5) buffers. The effects of temperature were determined across a range of temperatures from 20 to 70°C. Thermostability was determined by incubating 0.045 ug of purified enzyme at temperatures ranging from 25 to 70°C before testing the enzymatic activity. Relative activity was determined in comparison with the highest activity value measured among different temperatures or pH.

The effects of divalent ions were determined in 50 mM sodium phosphate buffer (pH 7) containing 5 mM various divalent ions (Mg^{2+}, Co^{2+}, Ni^{2+}, Cu^{2+}, Zn^{2+} or Ca^{2+}) or EDTA. Relative activity was determined in comparison with the enzyme activity measured in the sample without addition of divalent ions (i.e. buffer only).

The effects of incubation time was determined by incubating the purified Δ20*Pn*fucA in 50 mM sodium phosphate buffer (pH 7) up to 6 h. Relative activity was determined in comparison with the time point zero (i.e. t = 0 h).

To examine the enzyme's stability, the purified Δ20*Pn*fucA, maintained at a concentration of 2 mg/mL in sodium phosphate buffer (pH 7), underwent various storage conditions: 1) Stored at 4°C for 28 days. 2) Stored at -20°C for 28 days, followed by direct enzyme assay after thawing. 3) It underwent freeze-drying before being stored at 4°C for 28 days. Relative activity was determined in comparison with the enzyme activity measured with freshly purified enzyme on day 1.

The kinetics study was conducted with *p*NP-fucose concentrations ranging from 0.0625 to 16 mM. The K_m and V_{max} values were determined through nonlinear curve fitting utilizing the Michaelis-Menton model in Prism 10 (GraphPad, US).

Protein deglycosylation experiment

The deglycosylation by endo-β-N-acetylglucosaminidase (Endo-S2) was performed with 50 μg protein substrate and 5 μg Endo-S2 wild-type or mutant D184M enzymes in 50 mM sodium phosphate buffer (pH 7) at 37 °C for 3 hours. The complete removal of *N*-glycans was performed with 10 μg protein substrate and the Peptide-N-Glycosidase F (PNGase F, NEB) according to the manufacturer's recommendation. Fetuin from fetal bovine serum and BSA served as control proteins.

Endo-S2 mutant D184M glycosynthase activity experiment

To evaluate the glycosyntase efficiency of Endo-S2 mutant D184M, the hydrolysis reaction was monitored by incubating 100 μg original adalimumab (Hum_{Ori}) with 1 μg purified Endo-S2 mutant in a Tris buffer (50 mM Tris-HCl, 50 mM NaCl and 1 mM $CaCl_2$, pH 7.6) at 37 °C for 1 h, while the transglycosylation rate was determined by incubating 100 μg deglycosylated adalimumab with 1 μg purified Endo-S2 mutant in the presence of 10 mM sialyl-complex-type *N*-glycan-oxazoline (SCT-ox) under the same reaction conditions. To evaluate the SCT-ox concentration effects on the transglycosylation efficiency, 100 μg Hum_{Ori} was treated with 10 μg Endo-S2 mutant D184M and 10 μg Δ20*Pn*fucA in 50 mM Tris buffer (supplemented with 50 mM NaCl and 1 mM $CaCl_2$, pH 7.6) at 37 °C for 3 h. Then, the transglycosylation reaction was initiated by adding 0.1, 1

or 10 mM SCT-ox in the reaction mix, and the incubation continued at 37 °C for 6 h. The reaction products were taken after 1, 2, 4 and 6 hours, purified by Protein A chromatography, and then digested by thermolysin treatmenment. The *N*-glycan composition was determined by LC-ESI-MS analysis.

Determination of glycan-engineered adalimumab binding to FcγRIIIa

The binding activity was determined by ELISA assay. The FcγRIIIa V158 (GST-Fusion, AB Bioscience) (0.5μg/mL) was coated on a 96F MaxiSorp ELISA plate (NUNC) at 4 °C overnight in 100 μL of coating buffer composed of 50 mM carbonate-bicarbonate (pH 9.6). Then, the plate was washed three times with a washing buffer composed of PBS with 0.05% Tween 20 (PBST), and incubated at room temperature for 2 h after the addition of 200 μL of blocking buffer (2% BSA in PBS buffer). After the blocking step, the plate was washed with PBST before adding 200 μL of original adalimumab (Hum_{Ori}), deglycosylated and afucosylated adlimumab (Hum_{GlcNAc}) and homogeneous and afucosylated SCT-glycan adalimumab (Hum_{SCT}). Those sample were serially diluted in assay buffer (1% BSA in PBS) at concentrations ranging from 4000 to 0.49 ng/mL, and the plate was incubated at 4 °C overnight. On the third day, after washing the plate three times with PBST, 100 μL of Goat $(Fab')_2$ anti-human IgG Fcγ-HRP (Jackson ImmunoResearch Laboratories) diluted 1:10000 in assay buffer were added per well, and the plate was incubated at room temperature for 1 h. After a last washing step, 100 μL of TMB ELISA substrate (Abcam) were added. After at least 5 min in the dark, the color formation was stopped with 100 μL of stop solution (2N H_2SO_4), and the ELISA plate was read with a spectrophotometer (Varioskan Lux, Thermo Scientific) for the absorbance at

OD_{450}. The half maximal effective concentration (EC50) values were determined through nonlinear curve fitting utilizing the Sigmoidal 4PL model in Prism 10 (GraphPad, US).

Supplement Figure 1

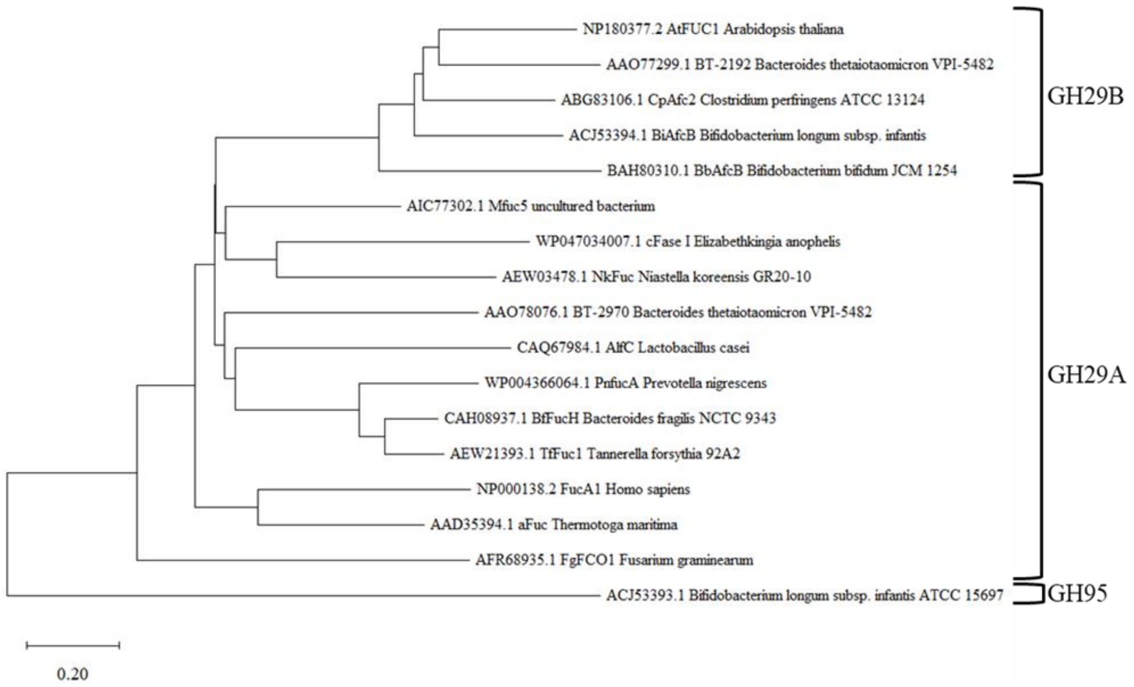

Figure S1: Phylogenic analysis of glycoside hydrolase family 29 (GH29) α-L-fucosidases. The phylogenetic tree was constructed using the Neighbor-Joining method in MEGA 11. Fucosidases that exhibit linkage selectivity towards α(1,3)- and α(1,4)-linked substrates were classified in the GH29B subgroup, while those with broad substrate specificity were classified in the GH29A subgroup. The bacterial GH 95 α-L-fucosidase from *Bifidobacterium longum* strain ATCC 15697 was used as the outgroup. All enzyme sequences were selected from CAZy[2], NCBI and PDB databases (listed in Table S3).

Supplement Figure 2

```
H. sapiens      WNSKDVGP-HRDLVGELGTALRKRNIRYGLYHS-LLEWFHPLYLLDKKNG---FKTQ---    199
T. maritima     FNSVKRGP-KRDLVGDLAKAVREAGLRFGVYYSGGLDWRFTTEPIRYPEDLSYIRPN---    197
L. casei        YNVYDATPFHRDIIGELAEACQKAGLKFGLYYSQDLDWHDPNG-----GGYKSNDVETAG    155
P. nigrescens   YNIVDATPFKRDVLKELANACHKQNIDLHIYYSI-LDWIREDYPIGRTGLYTGRKLK---    193

H. sapiens      -----------------HF-VSAKTMPELYDLVNSYK-PDLIWSDGEWECPDT----YWN    236
T. maritima     -----------------TYEYADYAYKQVMELVDLYL-PDVLWNDMGWPEKGK----ED-    234
L. casei        TTWDNSWDFPDEDQKNFDLCFDNKILPQIKEIMSNYGDIATAWFDVPMTLS------EAQ    209
P. nigrescens   --------------PNYG-TYFNFMKGQVSELLHNYGKVGAIWLDGYWDHDSDSIPFDWR    238

H. sapiens      STNFLSWLYNDSPVKDEVVVNDRWGQNCSCHHGGYYNCEDKFKPQSLPDH----------    286
T. maritima     LKYLFAYYYNKHP---EGSVNDRWGVPHWDFKTAEYH---VNYPGDLPGY----------    278
L. casei        SQTIYDTVRELQP---NCLINSRLGNGKYD-----FV---SLGDNEIPKNKEDMNKTDVD    258
P. nigrescens   MEEFYRYIHSIQP---ACLIGNNHHITPIDGED--FQ---MF-ERDLPGE----NKA---    282
```

Figure S2: Multiple sequence alignment of α-L-fucosidases. Identical amino acid residues among the four enzymes are highlighted in yellow. The catalytic nucleophilic and general acid-base residue are indicated in red and blue bold text, respectively. The alignment includes *Homo sapiens* α-L-fucosidase FucA1 (NP 000138.2), *Thermotoga maritima* α-L-fucosidase TM aFuc (AAD35394.1), *Lactobacillus casei* α-L-fucosidase AlfC (CAQ67984.1) and *Prevotella nigrescens* α-L-fucosidase *Pn*fucA (PDB: 7PLS) and was performed using Clustal Omega (https://www.ebi.ac.uk/Tools/msa/clustalo/).

Supplement Figure 3

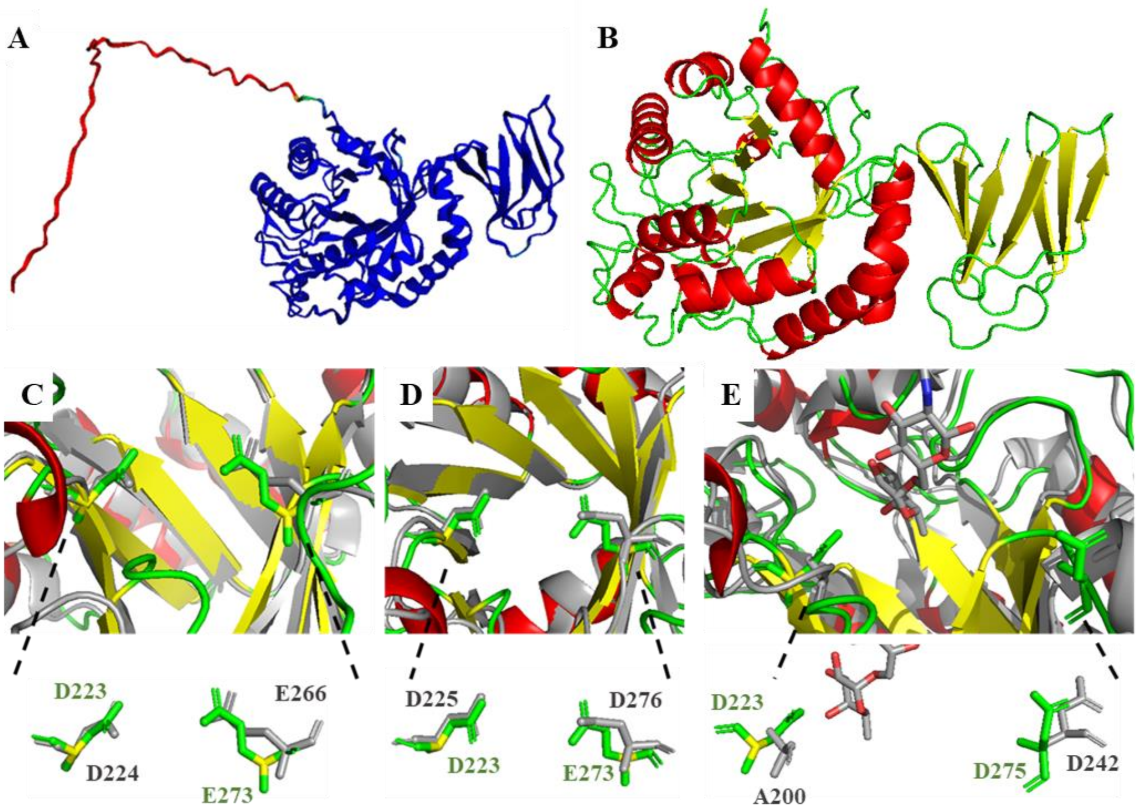

Figure S3: Identification of potential nucleophile and general acid/base residues in *Pn*fucA through protein structure alignment. (A) The 3D structure of *Pn*fucA was predicted with AlphaFold 2[3]. Amino acid residues with the confidence value or predicted Local Distance Difference Test (pLDDT) lower than 50 or higher than 90 were colored in red or in blue, respectively. (B) The predicted structure of *Pn*fucA exhibits a $(\beta/\alpha)_8$ barrel with an *N*-terminal β-sandwich domain. The α-helix, β-sheet, and loop regions are colored in red, yellow and green, respectively. (C to E) Structure superposition between *Pn*fucA and *Thermotoga maritina* TM aFuc (PDB:1HL9, C), *Homo sapien* FucA1 (PDB:7PLS, D) and *Lactobacillus casei* AlfC (PDB: 6OHE, E). *P*nfucA is in color, while the other α-L-fucosidases are in grey. The catalytic residues are represented by sticks.

Supplement Figure 4

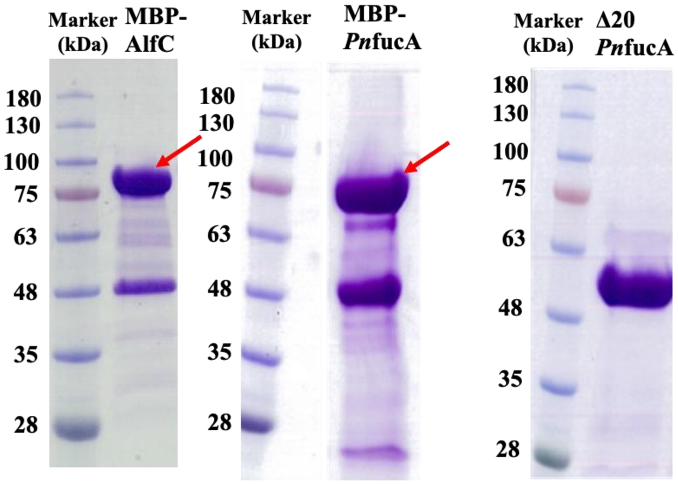

Figure S4: SDS-PAGE analysis of partially purified recombinant α-L-fucosidases AlfC and *Pn*fucA, and purified △20*Pn*fucA. The α-L-fucosidases AlfC from of *Lactobacillus casei* and *Pn*fucA from *Pretonella nigrescens*, expressed with an *N*-terminal maltose binding protein (MBP) fusion tag, were indicated with a red arrow.

Supplement Figure 5

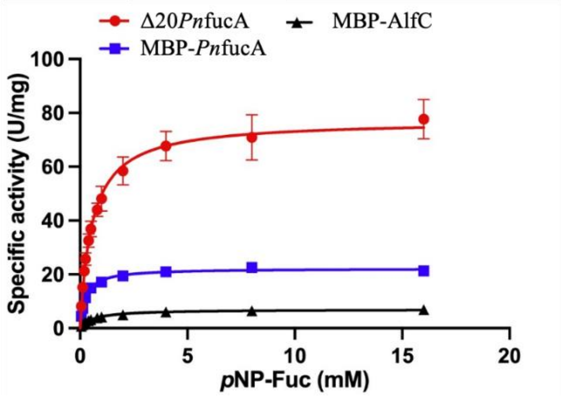

Figure S5: Kinetic study of recombinant α-L-fucosidases *Pn*fucA and AlfC. Enzyme reactions were performed using *p*-nitrophenyl-α-L-fucopyranoside (*p*NP-Fuc) at different concentrations in 50 mM sodium phosphate buffer (pH6.5). One enzyme unit (U) was defined as 1μmol of *p*NP produced per minute. Experiments were performed in triplicate, and error bars represent the standard deviation.

Supplement Figure 6

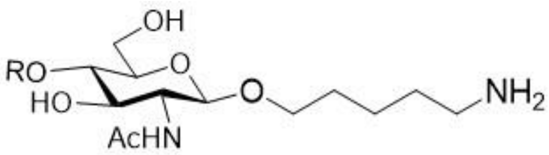

Figure S6: Chemical structure of the glycans with C-5 animo linker (C_5NH_2).

Supplement Figure 7

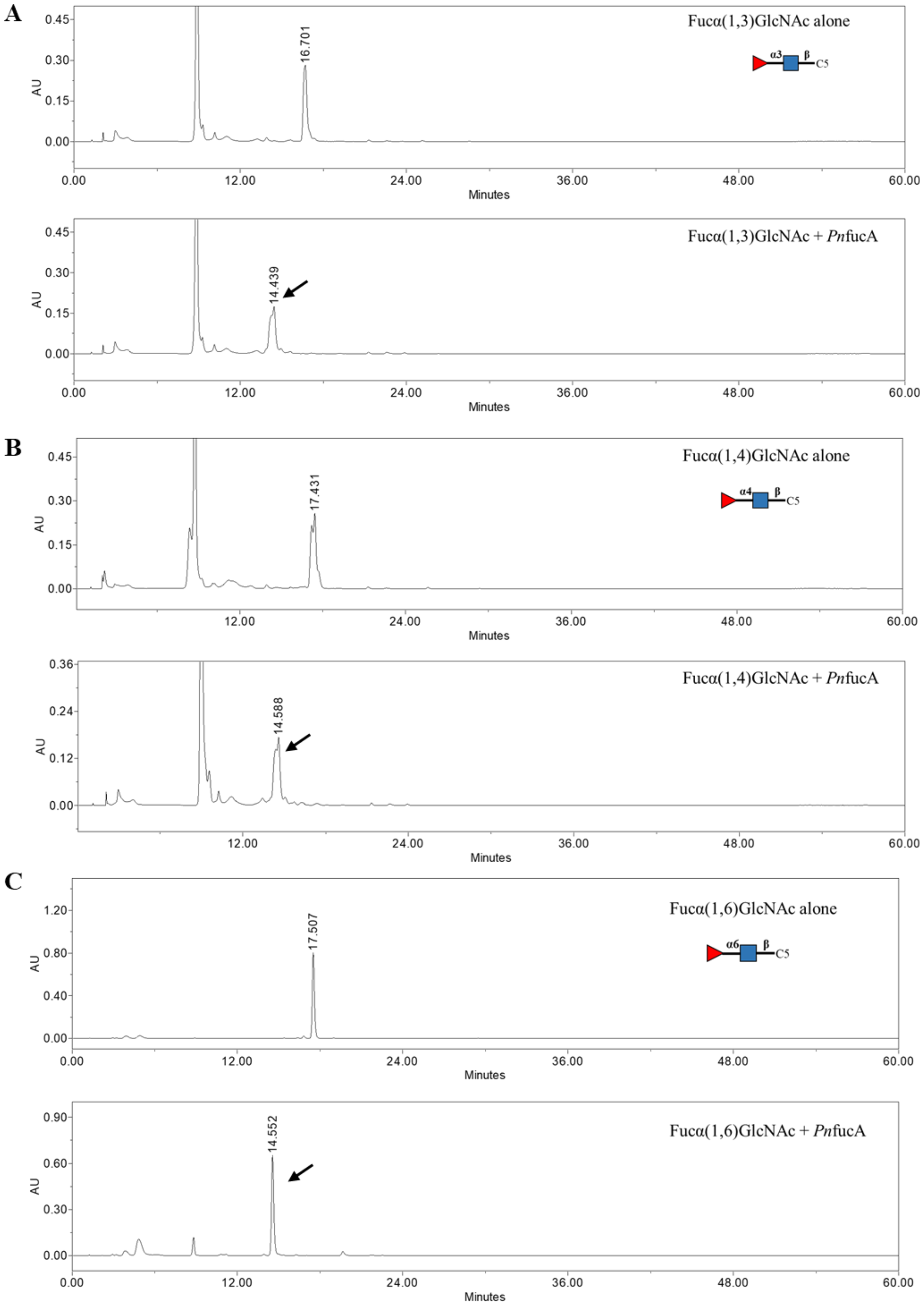

D

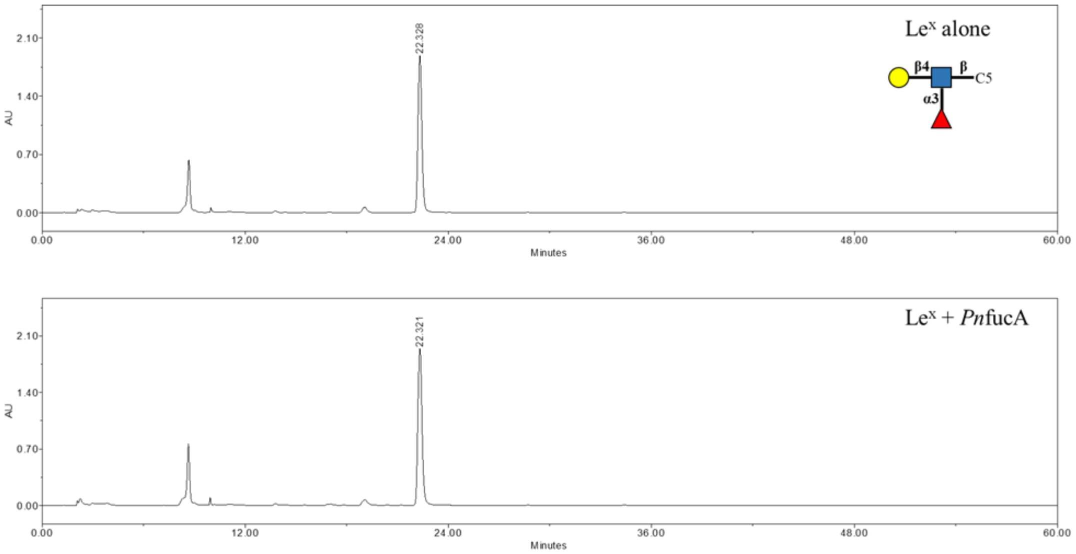
Le^x alone
β4
β
C5
α3
22.328
Le^x + *Pn*fucA
22.321
AU
Minutes

E

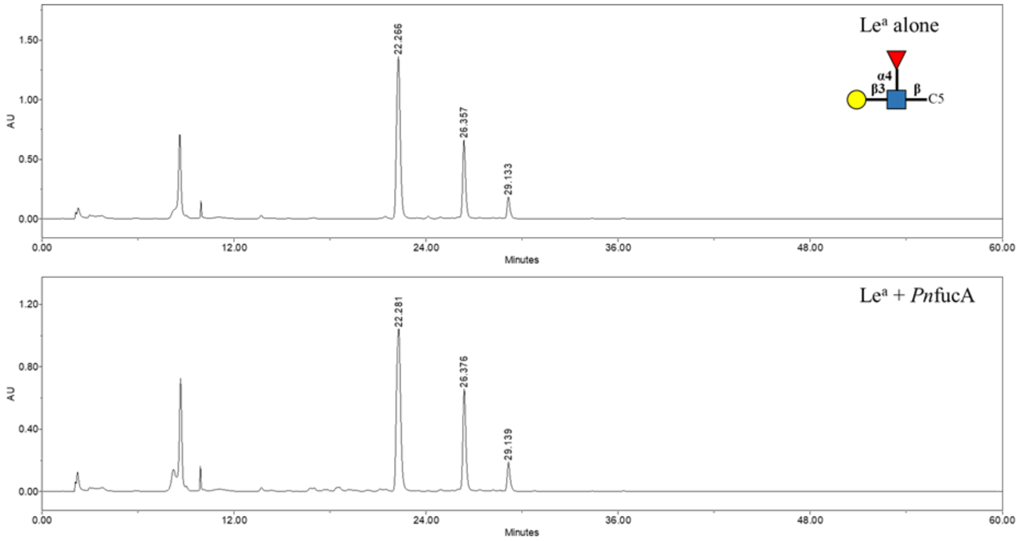
Le^a alone
α4
β3
β
C5
22.266
26.357
29.133
Le^a + *Pn*fucA
22.281
26.376
29.139
AU
Minutes

F

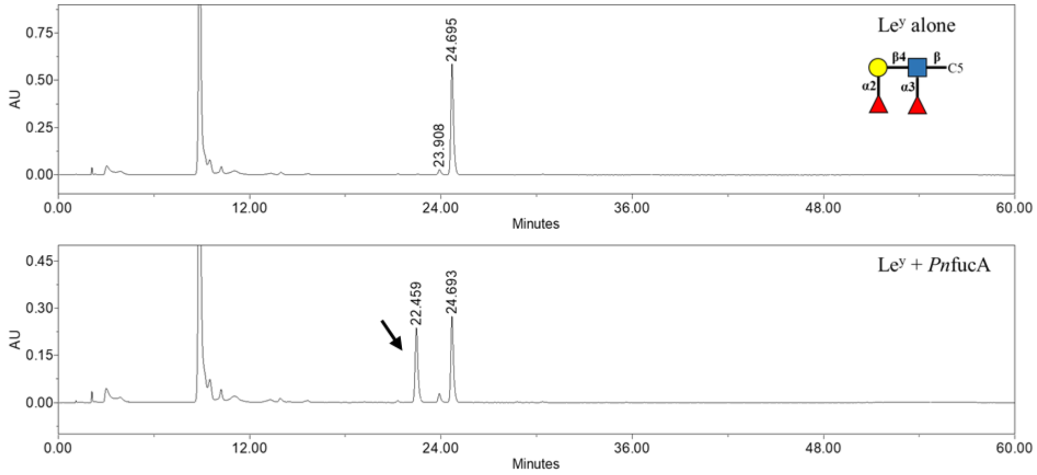
Le^y alone
β4
β
C5
α2
α3
24.695
23.908
Le^y + *Pn*fucA
22.459
24.693
AU
Minutes

G

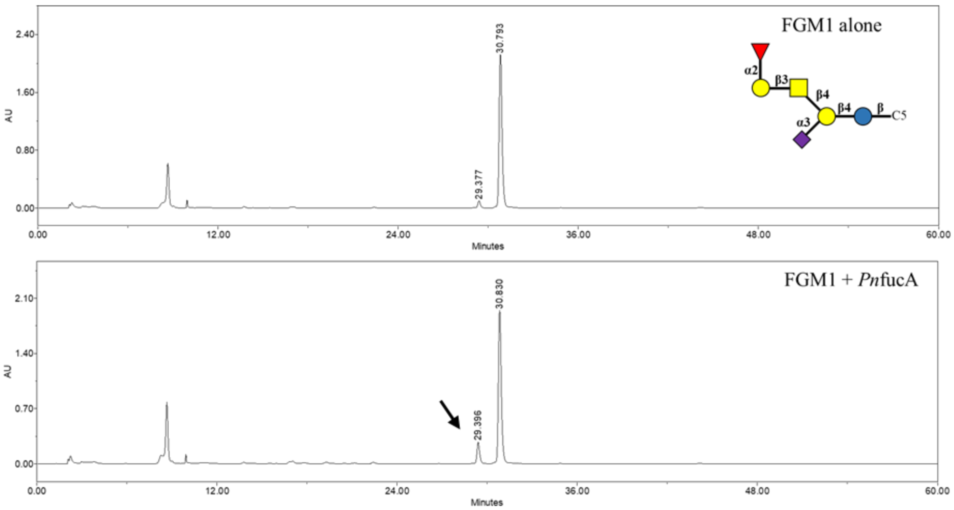
FGM1 alone
30.793
29.377
AU
Minutes
FGM1 + PnfucA
30.830
29.396
α2
β3
β4
α3
β4
β
C5

H

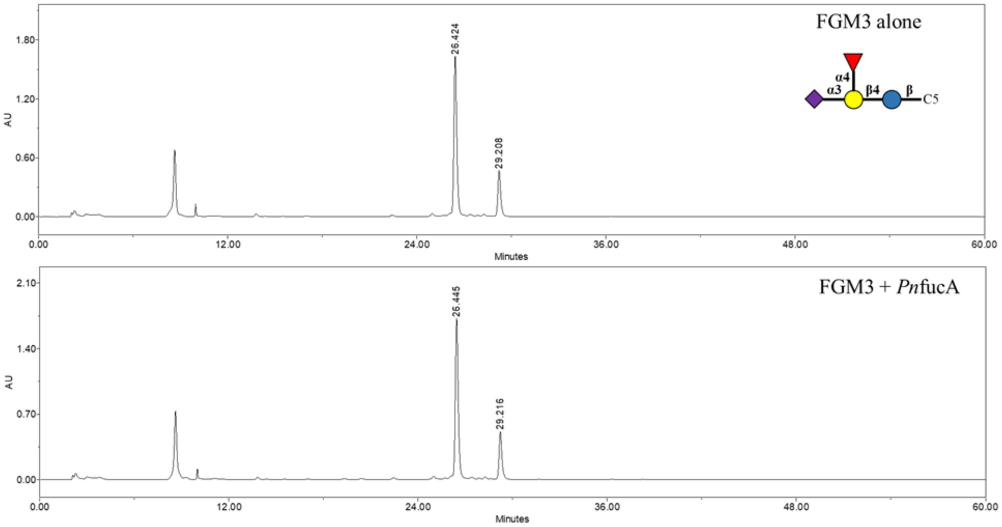
FGM3 alone
26.424
29.208
AU
Minutes
FGM3 + PnfucA
26.445
29.216
α4
α3
β4
β
C5

I

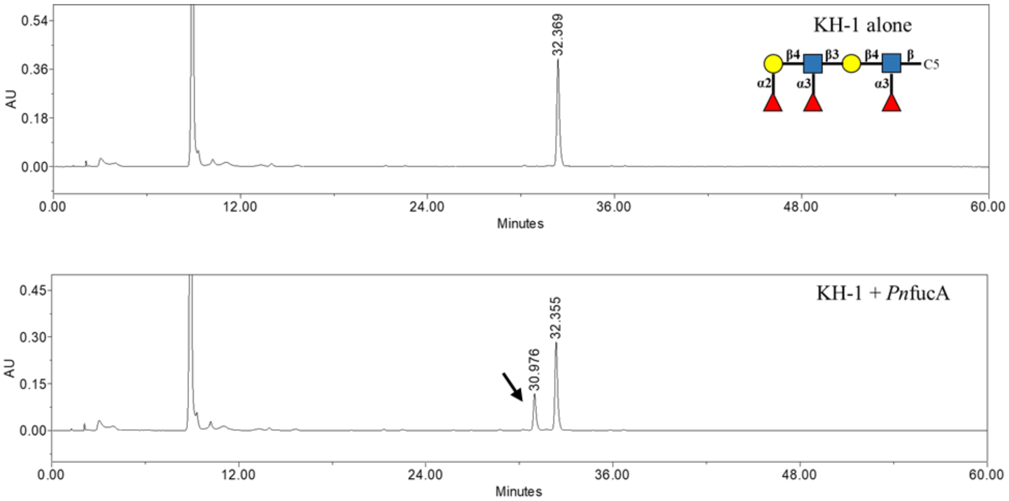
KH-1 alone
32.369
AU
Minutes
KH-1 + PnfucA
30.976
32.355
β4
β3
β4
β
C5
α2
α3
α3

J

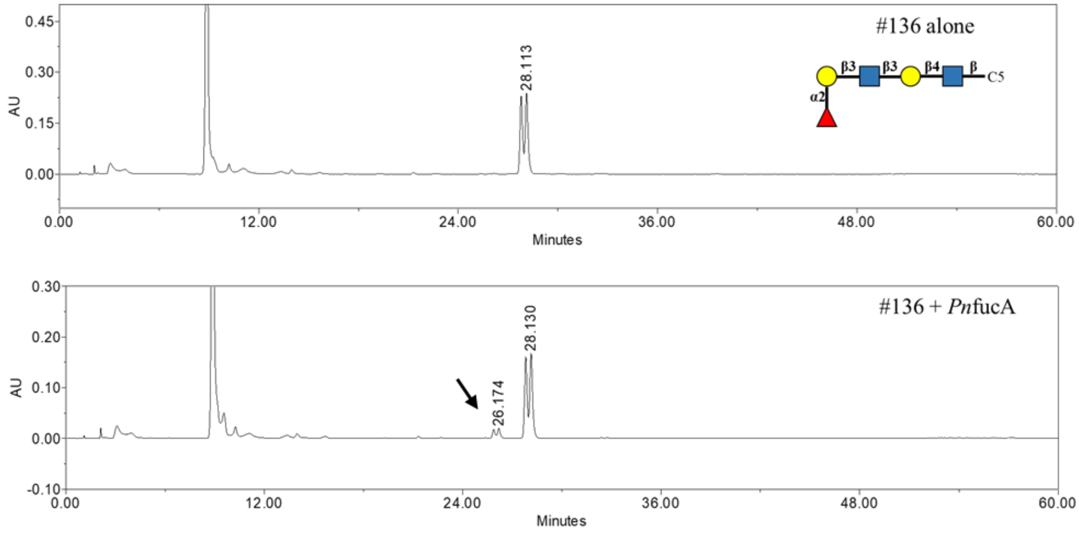
#136 alone
28.113
Minutes
AU
#136 + *Pn*fucA
26.174
28.130
α2
β3
β4
β
C5

K

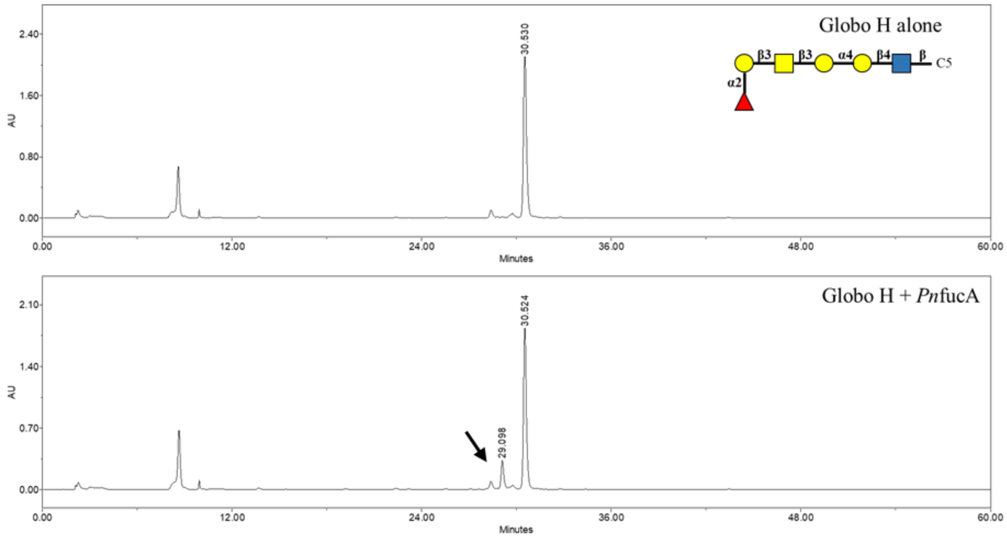
Globo H alone
30.530
Globo H + *Pn*fucA
29.098
30.524
α2
β3
α4
β4
β
C5
AU
Minutes

L

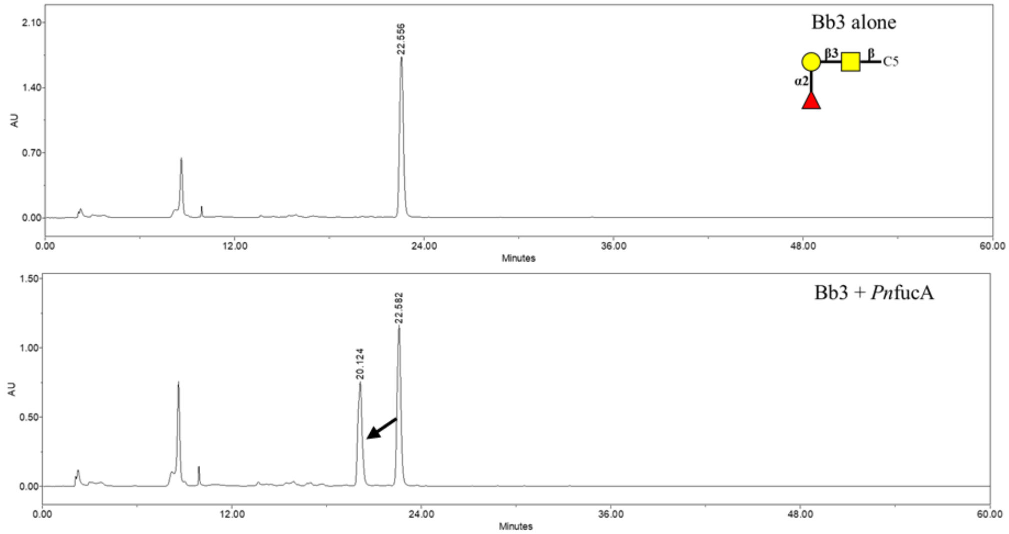
Bb3 alone
22.556
Bb3 + *Pn*fucA
20.124
22.582
α2
β3
β
C5
AU
Minutes

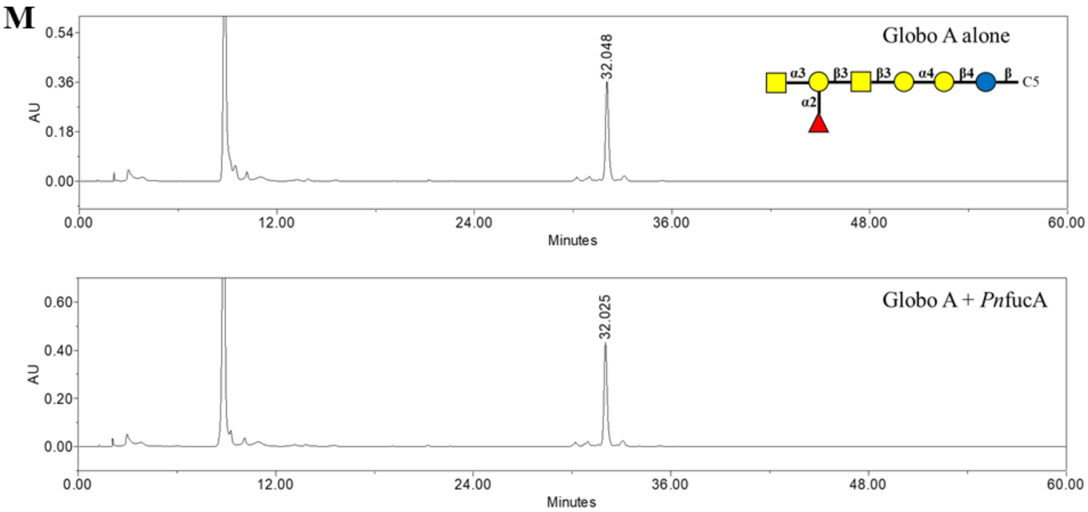

Figure S7: HPLC analysis of fucosylated substrates hydrolyzed by Δ20*Pn*fucA. Substrates include: Fucα(1,3)GlcNAc (A); Fucα(1,4)GlcNAc (B); Fucα(1,6)GlcNAc (C); Le^x (D); Le^a (E); Le^y (F); FGM1 (G); FGM3 (H); KH-1 (I); glycan #136 (J); Globo H (K); Bb3 (L) and Globo A (M). Peaks corresponding to hydrolysis products are marked by arrows.

Supplement Figure 8

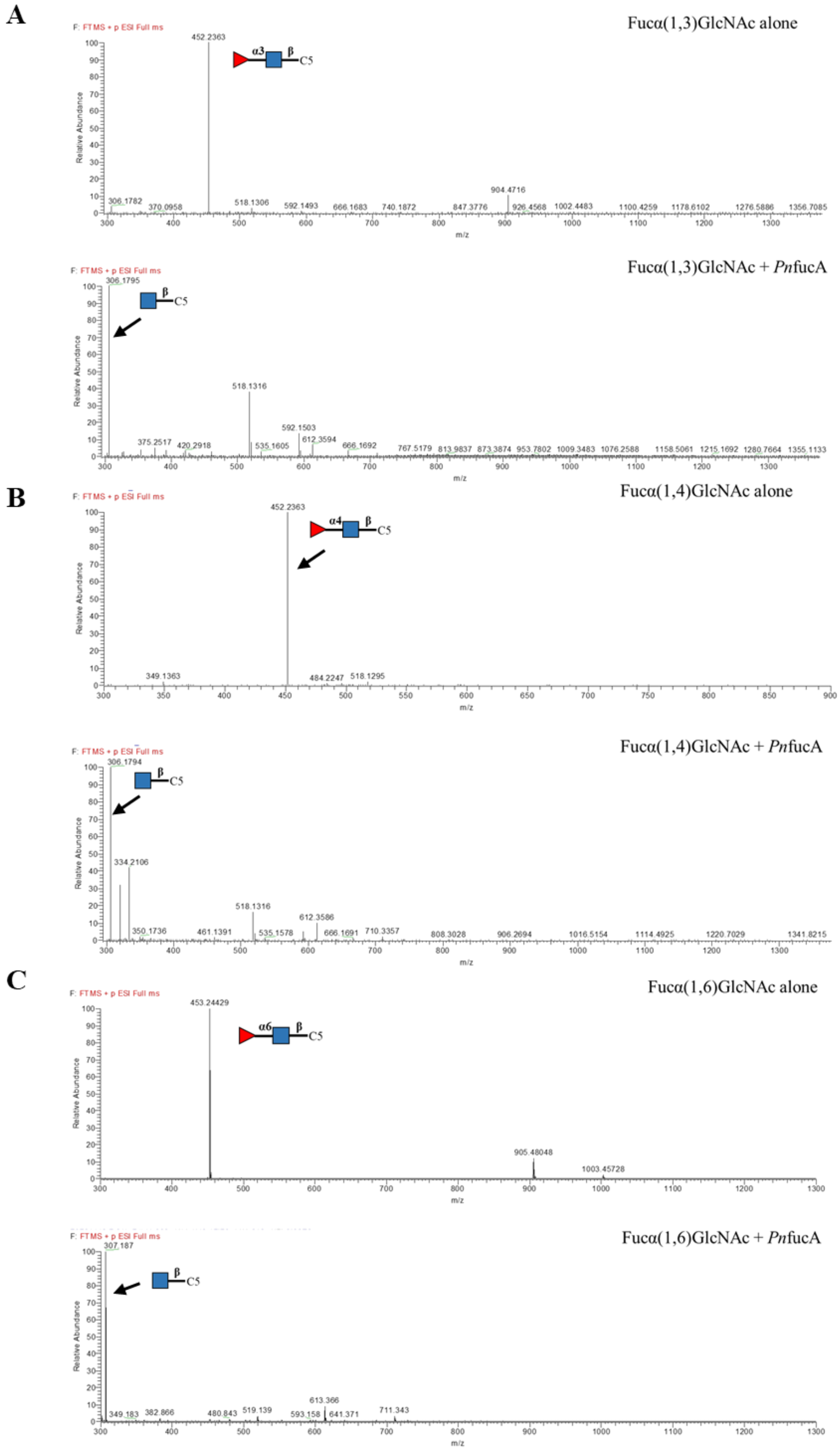

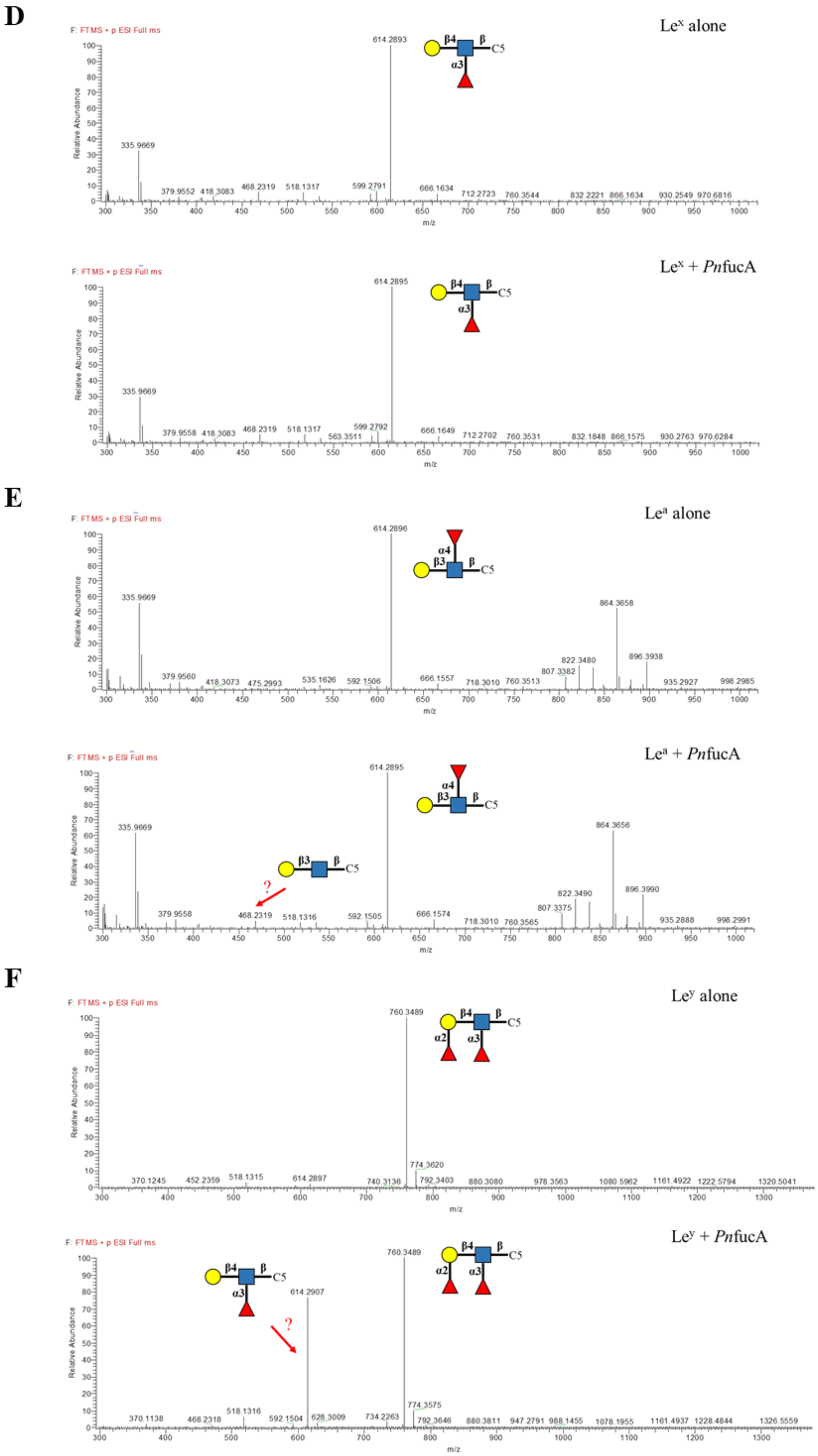
D
Le^x alone
F: FTMS + p ESI Full ms
614.2893
335.9669
Relative Abundance
m/z
C5
Le^x + PnfucA
614.2895
335.9669
E
Le^a alone
614.2896
335.9669
864.3658
896.3938
822.3480
Le^a + PnfucA
614.2895
335.9669
864.3656
896.3990
822.3490
468.2319
?
F
Le^y alone
760.3489
774.3620
Le^y + PnfucA
760.3489
614.2907
774.3575
518.1316
?

G

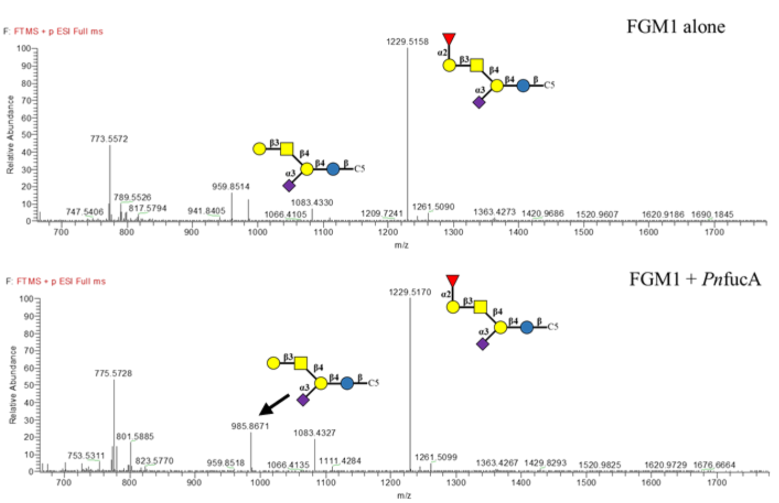
FGM1 alone
F: FTMS + p ESI Full ms
1229.5158
773.5572
959.8514
1083.4330
Relative Abundance
m/z
FGM1 + *Pn*fucA
F: FTMS + p ESI Full ms
1229.5170
775.5728
985.8671
1083.4327
Relative Abundance
m/z

H

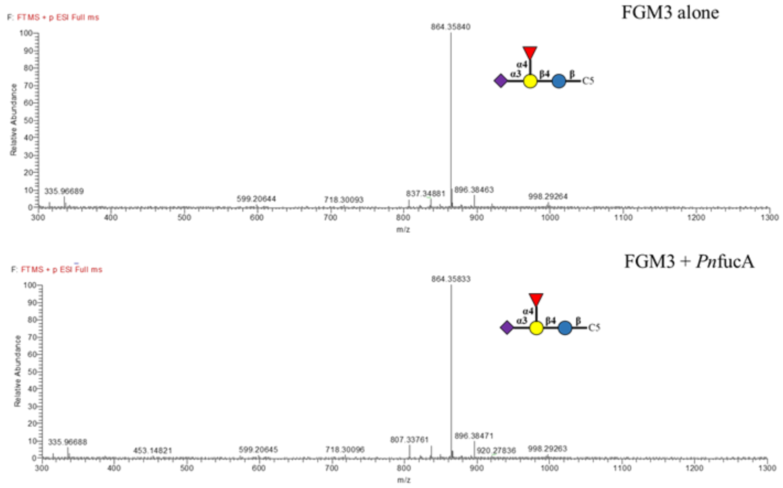
FGM3 alone
F: FTMS + p ESI Full ms
864.35840
896.38463
Relative Abundance
m/z
FGM3 + *Pn*fucA
F: FTMS + p ESI Full ms
864.35833
896.38471
Relative Abundance
m/z

I

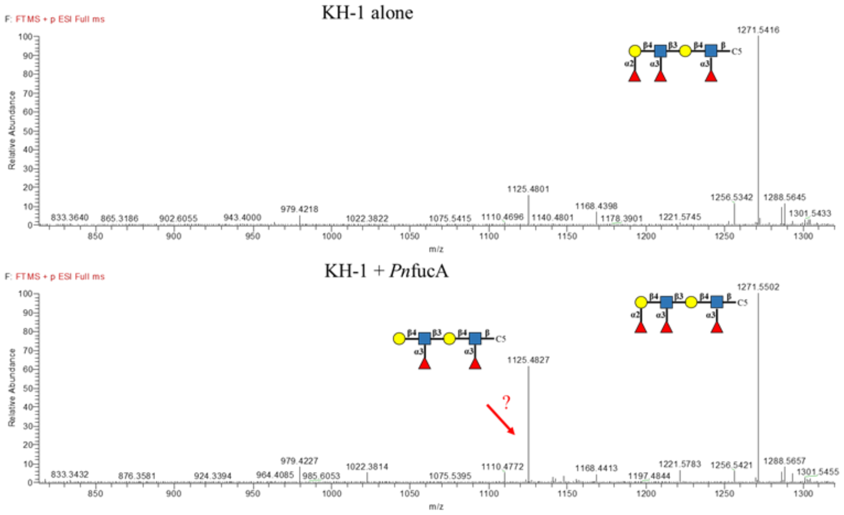
KH-1 alone
F: FTMS + p ESI Full ms
1271.5416
1125.4801
Relative Abundance
m/z
KH-1 + *Pn*fucA
F: FTMS + p ESI Full ms
1271.5502
1125.4827
?
Relative Abundance
m/z

J

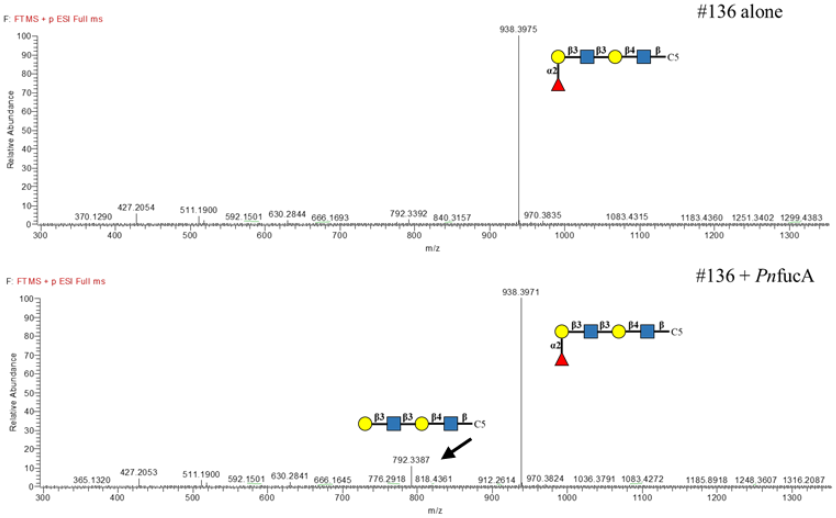
#136 alone
F: FTMS + p ESI Full ms
938.3975
Relative Abundance
m/z
#136 + *Pn*fucA
F: FTMS + p ESI Full ms
938.3971
792.3387
C5

K

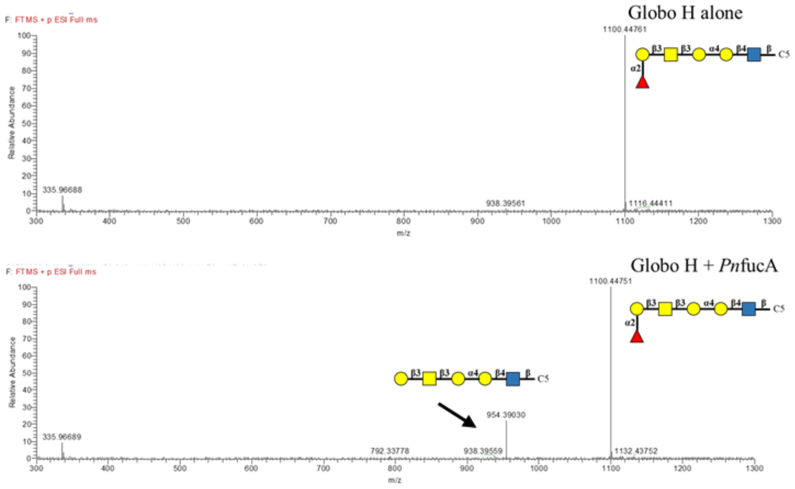
Globo H alone
F: FTMS + p ESI Full ms
1100.44761
335.96688
938.39561
1116.44411
Relative Abundance
m/z
Globo H + *Pn*fucA
F: FTMS + p ESI Full ms
1100.44751
954.39030
335.96689
792.33778
938.39559
1132.43752
C5

L

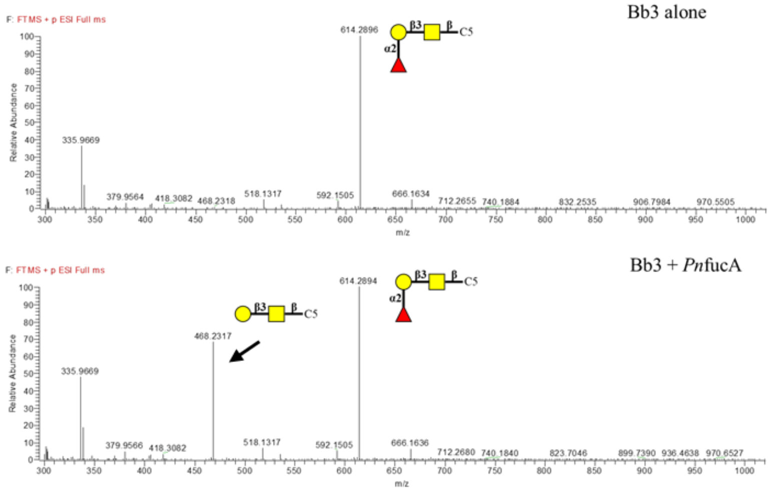
Bb3 alone
F: FTMS + p ESI Full ms
614.2896
335.9669
Relative Abundance
m/z
Bb3 + *Pn*fucA
F: FTMS + p ESI Full ms
614.2894
468.2317
335.9669
C5

M

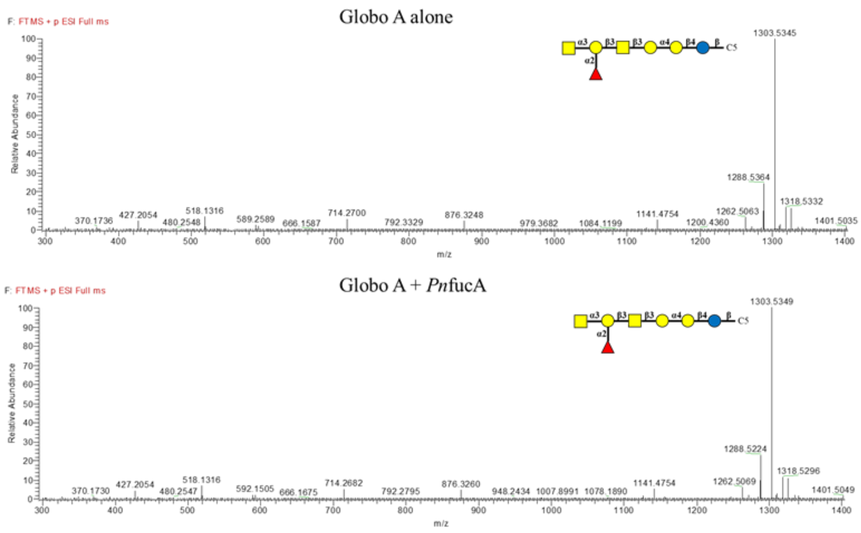
Globo A alone
F: FTMS + p ESI Full ms
Relative Abundance
m/z
1303.5345
1288.5364
1318.5332
1262.5063
1141.4754
1200.4360
1401.5035
370.1736
427.2054
480.2548
518.1316
589.2589
666.1587
714.2700
792.3329
876.3248
979.3682
1084.1199
α3 β3 β3 α4 β4 β C5 α2
Globo A + *Pn*fucA
1303.5349
1288.5224
1318.5296
1262.5069
1141.4754
1401.5049
370.1730
427.2054
480.2547
518.1316
592.1505
666.1675
714.2682
792.2795
876.3260
948.2434
1007.8991
1078.1890

N

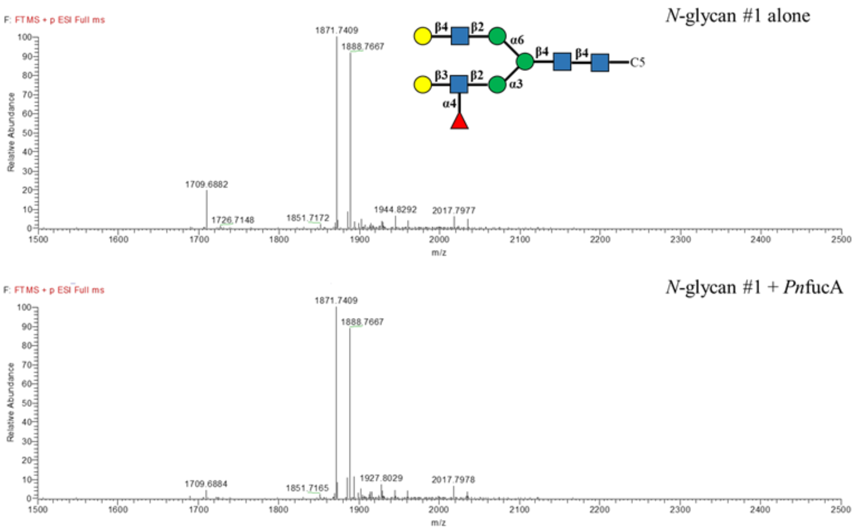
N-glycan #1 alone
F: FTMS + p ESI Full ms
Relative Abundance
m/z
1871.7409
1888.7667
1709.6882
1726.7148
1851.7172
1944.8292
2017.7977
β4 β2 α6 β4 β4 C5 β3 β2 α3 α4
N-glycan #1 + *Pn*fucA
1871.7409
1888.7667
1709.6884
1851.7165
1927.8029
2017.7978

O

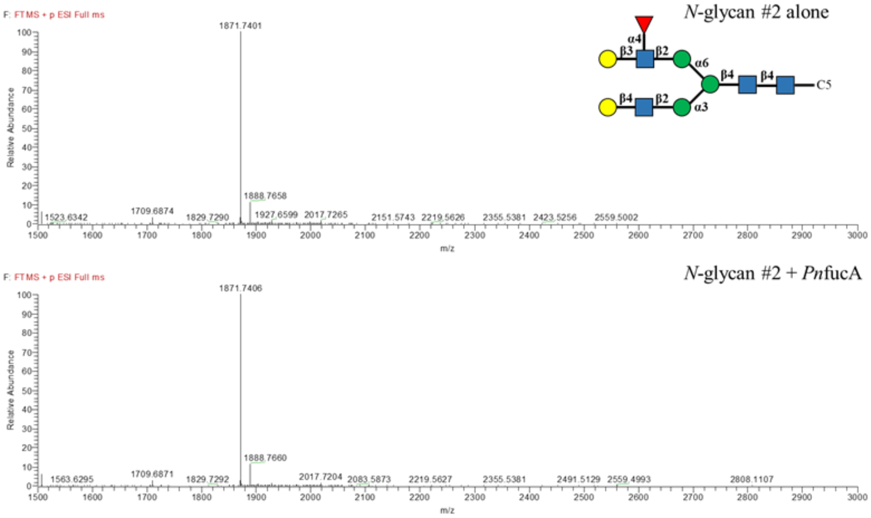
N-glycan #2 alone
F: FTMS + p ESI Full ms
Relative Abundance
m/z
1871.7401
1888.7658
1523.6342
1709.6874
1829.7290
1927.6599
2017.7265
2151.5743
2219.5626
2355.5381
2423.5256
2559.5002
α4 β3 β2 α6 β4 β4 C5 β4 β2 α3
N-glycan #2 + *Pn*fucA
1871.7406
1888.7660
1563.6295
1709.6871
1829.7292
2017.7204
2083.5873
2219.5627
2355.5381
2491.5129
2559.4993
2808.1107

P

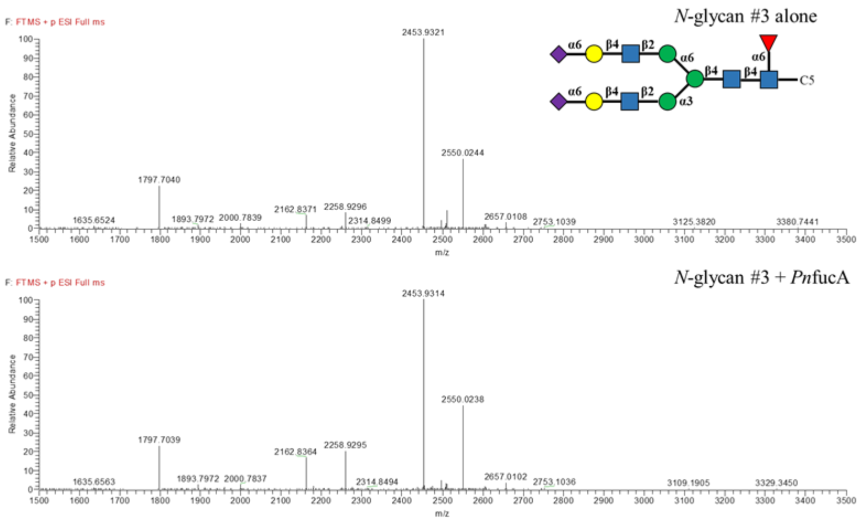

Figure S8: LC-MS analysis of fucosylated substrates hydrolyzed by Δ20*Pn*fucA. Substrates: Fucα(1,3)GlcNAc (A); Fucα(1,4)GlcNAc (B); Fucα(1,6)GlcNAc (C); Le^x (D); Le^a (E); Le^y (F); FGM1 (G); FGM3 (H); KH-1 (I); glycan #136 (J); Globo H (K); Bb3 (L); Globo A (M); fucosylated N-glycans #1, #2 and #3 (N-P). Peaks corresponding to hydrolysis products are marked by arrows (potential hydrolysis product in red).

Supplement Table 1

Table S1: Substrate specificity of some GH29A α-L-fucosidases presented in the phylogenetic tree.

**Enzyme**	**Substrate**							**Reference**
	2'-FL	3-FL	Le^x	Le^a	Fucα(1,3)GlcNAc	Fucα(1,4)GlcNAc	Fucα(1,6)GlcNAc	
AlfC	-	-	-	-	+	+	+	4
BT-2970	Trace	-	+	+	-	-	-	5
FgFCO1	+	-	x	-	x	x	x	6
TfFuc1	+	-	-	-	+	+	+	7, 8
Mfuc5	+	+	-	+	-	+	+	8, 9
cFase I	-	+	+	-	x	x	-	10
NkFuc	-	-	-	-	-	-	+	8
Bf FucH	x	x	-	-	x	x	+	11
Pn fucA	x	x	-	-	+	+	+	This study

2'-FL: 2'-fucosyllactose; 3-FL: 3-fucosyllactose

x: not tested

Supplement Table 2

N-glycan structure	*N*-glycan composition	*N*-linked peptide	Mass	LC Retention Time (min)
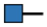	GlcNAc(1)	VHNAKTKPREEQYN	1915.93882	18.0659
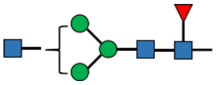	GlcNAc(3)Man(3)Fuc(1) (G0F-N)	VHNAKTKPREEQYN	2954.31392	18.4542
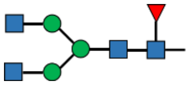	GlcNAc(4)Man(3)Fuc(1) (G0F)	VHNAKTKPREEQYN	3157.39332	18.166
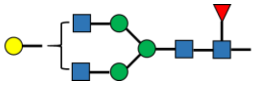	GlcNAc(4)Man(4)Fuc(1) (G1F)	VHNAKTKPREEQYN	3319.44612	18.237
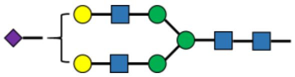	GlcNAc(4)Man(5)NeuAc(1) (G2F+NANA)	VHNAKTKPREEQYN	3626.53642	17.9867
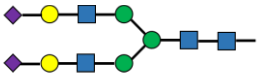	GlcNAc(4)Man(5)NeuAc(2) (G2F+2NANA)	VHNAKTKPREEQYN	3917.63182	18.1566

Table S2: Composition, mass and retention time of *N*-glycosylated peptides from thermolysin-digested adalimumab, detected by the LC-MS method. Abbreviations: mono-*N*-acetylglucosaminylated (G0-N), agalactosylated (G0), mono-galactosylated (G1), mono-sialylated (G2-NANA) and di-sialylated (G2+2NANA) complex-type *N*-glycans. F: with core-fucosylation.

Supplement Figure 9

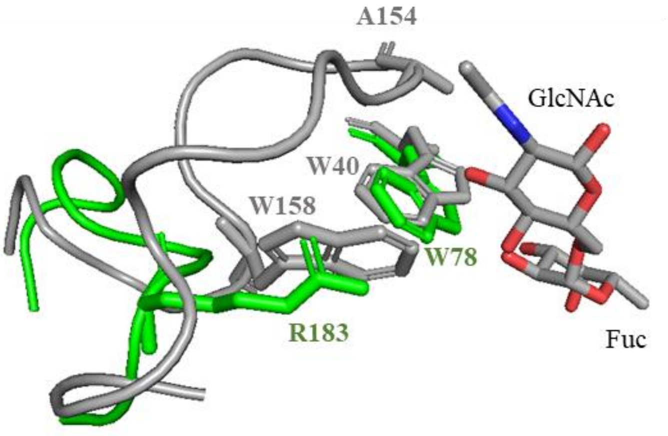

Figure S9: Representation of the Fucα(1,6)GlcNAc binding pocket in *L. casei* AlfC. Amino acids involved in substrate binding are represented in sticks. Loops were formed by residues G145-D163 in AlfC and I181-R190 in the *Pn*fucA. AlfC and *Pn*fucA are colored in grey and green, respectively.

Supplement Figure 10

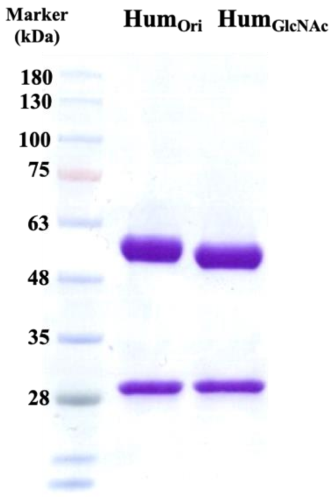

Figure S10: SDS-PAGE analysis of purified adalimumab (Hum$_{Ori}$) and deglycosylated/ afucosylated adalimumab (Hum$_{GlcNAc}$) after Protein A purification.

Supplement Figure 11

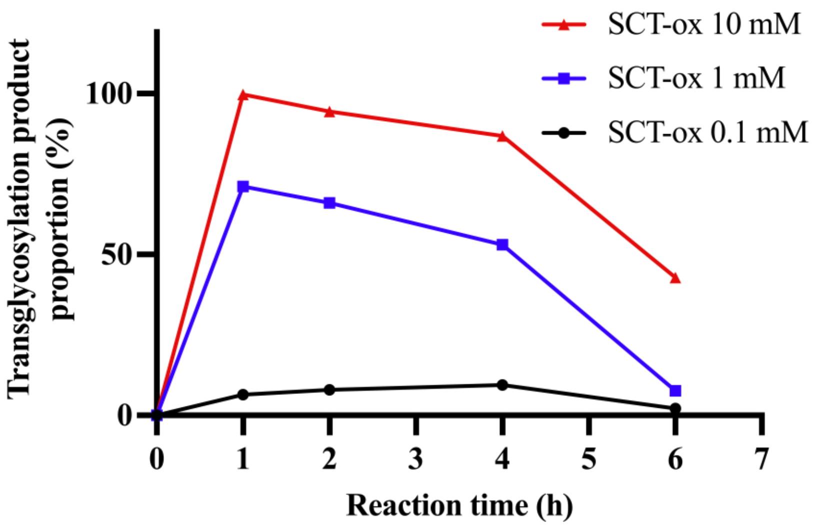

Figure S11: Moniting the transglycosylation products (Hum_{SCT}) in the presence of different SCT-ox concentrations. The Hum_{GlcNAc} was generated by treating 100 μg commercial adalimumab (Hum_{Ori}) with 10 μg Endo-S2 mutant D184M and 10 μg Δ20*Pn*fucA. The transglycosylation of Hum_{GlcNAc} was performed by adding 0.1, 1 and 10 mM SCT-ox at t = 0 h. The proportion of transglycosylation product was monitored at 1, 2, 4, and 6 hours of reaction.

.

Supplement Table 3

Accession number	Organism
NP_180377.2	AtFUC1 *Arabidopsis thaliana*
CAH08937.1	BfFucH *Bacteroides fragilis* NCTC 9343
AAO77299.1	BT-2192 *Bacteroides thetaiotaomicron* VPI-5482
AAO78076.1	BT-2970 *Bacteroides thetaiotaomicron* VPI-5482
BAH80310.1	BbAfcB *Bifidobacterium bifidum* JCM 1254
ACJ53394.1	BiAfcB *Bifidobacterium longum subsp. infantis*
ACJ53393.1	*Bifidobacterium longum subsp. infantis* ATCC 15697
ABG83106.1	CpAfc2 *Clostridium perfringens* ATCC 13124
WP_047034007.1	cFase I *Elizabethkingia anophelis*
AFR68935.1	FgFCO1 *Fusarium graminearum*
NP_000138.2	FucA1 *Homo sapiens*
CAQ67984.1	AlfC *Lactobacillus casei*
WP_004366064.1	*Pn* fucA *Prevotella nigrescens*
AEW21393.1	TfFuc1 *Tannerella forsythia* 92A2
AAD35394.1	aFuc *Thermotoga maritima*
AIC77302.1	Mfuc5 uncultured bacterium
AEW03478.1	NkFuc *Niastella koreensis* GR20-10

Table S3: List of α-L-fucosidases mentioned in the Figure S1.

1. Bradford, M. M., A rapid and sensitive method for the quantitation of microgram quantities of protein utilizing the principle of protein-dye binding. *Anal. Biochem.* **1976,** *72* (1), 248-254.

2. Drula, E.; Garron, M.-L.; Dogan, S.; Lombard, V.; Henrissat, B.; Terrapon, N., The carbohydrate-active enzyme database: functions and literature. *Nucleic Acids Res.* **2022**, *50* (D1), D571-D577.

3. Jumper, J.; Evans, R.; Pritzel, A.; Green, T.; Figurnov, M.; Ronneberger, O.; Tunyasuvunakool, K.; Bates, R.; Žídek, A.; Potapenko, A.; Bridgland, A.; Meyer, C.; Kohl, S. A. A.; Ballard, A. J.; Cowie, A.; Romera-Paredes, B.; Nikolov, S.; Jain, R.; Adler, J.; Back, T.; Petersen, S.; Reiman, D.; Clancy, E.; Zielinski, M.; Steinegger, M.; Pacholska, M.; Berghammer, T.; Bodenstein, S.; Silver, D.; Vinyals, O.; Senior, A. W.; Kavukcuoglu, K.; Kohli, P.; Hassabis, D., Highly accurate protein structure prediction with AlphaFold. *Nature* **2021,** *596* (7873), 583-589.

4. Rodríguez-Díaz, J.; Monedero, V.; Yebra, M. J., Utilization of natural fucosylated oligosaccharides by three novel α-L-fucosidases from a probiotic *Lactobacillus casei* strain. *Appl. Environ. Microbiol.* **2011**, *77* (2), 703-705.

5. Sakurama, H.; Tsutsumi, E.; Ashida, H.; Katayama, T.; Yamamoto, K.; Kumagai, H., Differences in the substrate specificities and active-site structures of two α-L-fucosidases (glycoside hydrolase family 29) from *Bacteroides thetaiotaomicron*. *Biosci. Biotechnol. Biochem.* **2012**, *76* (5), 1022-1024.

6. Cao, H.; Walton, J. D.; Brumm, P.; Phillips, G. N., Structure and substrate specificity of a eukaryotic fucosidase from *Fusarium graminearum*. *J. Biol. Chem.* **2014**, *289* (37), 25624-25638.

7. Megson, Z. A.; Koerdt, A.; Schuster, H.; Ludwig, R.; Janesch, B.; Frey, A.; Naylor, K.; Wilson, I. B.; Stafford, G. P.; Messner, P.; Schäffer, C., Characterization of an α-L-fucosidase from the periodontal pathogen *Tannerella forsythia*. *Virulence* **2015**, *6* (3), 282-292.

8. Perna, V. N.; Barrett, K.; Meyer, A. S.; Zeuner, B., Substrate specificity and transglycosylation capacity of α-L-fucosidases across GH29 assessed by bioinformatics-assisted selection of functional diversity. *Glycobiology* **2023**, *33* (5), 396-410.

9. Lezyk, M.; Jers, C.; Kjaerulff, L.; Gotfredsen, C. H.; Mikkelsen, M. D.; Mikkelsen, J. D., Novel α-L-fucosidases from a soil metagenome for production of fucosylated human milk oligosaccharides. *PLoS One* **2016**, *11* (1), e0147438.

10. Li, T.; Li, M.; Hou, L.; Guo, Y.; Wang, L.; Sun, G.; Chen, L., Identification and characterization of a core fucosidase from the bacterium *Elizabethkingia meningoseptica*. *J. Biol. Chem.* **2018**, *293* (4), 1243-1258.

11. Tsai, T. I.; Li, S. T.; Liu, C. P.; Chen, K. Y.; Shivatare, S. S.; Lin, C. W.; Liao, S. F.; Lin, C. W.; Hsu, T. L.; Wu, Y. T.; Tsai, M. H.; Lai, M. Y.; Lin, N. H.; Wu, C. Y.; Wong, C. H., An effective bacterial fucosidase for glycoprotein remodeling. *ACS Chem. Biol.* **2017**, *12* (1), 63-72.
